# Aflatoxins: Producing-Molds, Structure, Health Issues and Incidence in Southeast Asian and Sub-Saharan African Countries

**DOI:** 10.3390/ijerph17041215

**Published:** 2020-02-13

**Authors:** Noreddine Benkerroum

**Affiliations:** Department of Food Science and Agricultural Chemistry, MacDonald Campus, McGill University, 21111 Lakeshore, Ste Anne de Bellevue, QC H9X 3V9, Canada; n.benkerroum@gmail.com; Tel.: +1-514-652-4945

**Keywords:** aflatoxins, incidence, toxicity, risk assessment, biocontrol, atoxigenic *A. flavus*

## Abstract

This review aims to update the main aspects of aflatoxin production, occurrence and incidence in selected countries, and associated aflatoxicosis outbreaks. Means to reduce aflatoxin incidence in crops were also presented, with an emphasis on the environmentally-friendly technology using atoxigenic strains of *Aspergillus flavus*. Aflatoxins are unavoidable widespread natural contaminants of foods and feeds with serious impacts on health, agricultural and livestock productivity, and food safety. They are secondary metabolites produced by *Aspergillus* species distributed on three main sections of the genus (section *Flavi*, section *Ochraceorosei*, and section *Nidulantes*). Poor economic status of a country exacerbates the risk and the extent of crop contamination due to faulty storage conditions that are usually suitable for mold growth and mycotoxin production: temperature of 22 to 29 °C and water activity of 0.90 to 0.99. This situation paralleled the prevalence of high liver cancer and the occasional acute aflatoxicosis episodes that have been associated with these regions. Risk assessment studies revealed that Southeast Asian (SEA) and Sub-Saharan African (SSA) countries remain at high risk and that, apart from the regulatory standards revision to be more restrictive, other actions to prevent or decontaminate crops are to be taken for adequate public health protection. Indeed, a review of publications on the incidence of aflatoxins in selected foods and feeds from countries whose crops are classically known for their highest contamination with aflatoxins, reveals that despite the intensive efforts made to reduce such an incidence, there has been no clear tendency, with the possible exception of South Africa, towards sustained improvements. Nonetheless, a global risk assessment of the new situation regarding crop contamination with aflatoxins by international organizations with the required expertise is suggested to appraise where we stand presently.

## 1. Introduction

Mycotoxins are among the microbial toxins of most concern to public health, and they represent a barrier to a wider international trade of agri-food products and an important obstacle in the face of the harmonization of regulatory standards globally, as was discussed earlier [1]. They are produced by various mould species as low-molecular-weight non-immunogenic secondary metabolites whose occurrence has been reported in virtually all foods and feeds [2,3]. Mycotoxins are believed to provide the producing-strains with competitive advantage over the microbiota of the same niche [4] and a protective measure against predatory insects [5]. In plants they have been suggested to help invading the host tissues by promoting cell death and tissue necrosis [6]. Currently, there are more than 450 different known types of mycotoxins and their metabolites, which have been associated with toxicological effects of varying severity degrees spanning from mild gastroenteritis to deadly cancer diseases [7,8]. Aflatoxins produced mainly by *Aspergillus* species are the most toxic mycotoxins eliciting acute and chronic toxicities, the most severe and notorious of which are genotoxicity, mutagenicity, and immunotoxicity. Their toxicological status as human carcinogens is now beyond doubt, and it has been recognized by the International Agency for Research on Cancer (IARC) [9].

Although aflatoxins are of a global concern, their negative impact on health, economy, and social life is greater in developing countries located in the tropical and sub-tropical regions. Agricultural products from SSA countries, e.g., Gambia, Uganda, Kenya, and Tanzania, and SEA countries, e.g., China, Thailand, Vietnam, and Indonesia, have classically been associated with the highest incidence of aflatoxins, which paralleled the highest incidence of hepatocellular carcinoma and the occurrence of acute aflatoxicosis outbreak episodes in the region [10]. As matter of fact, these regions have been the primary destination for scientists to carry out epidemiological studies on the relationship between the dietary exposure to aflatoxins and liver cancer, which contributed greatly to the establishment of aflatoxins as an aetiological factor of the disease in humans. Four major types of aflatoxins [aflatoxin B1, aflatoxin B2 (AFB1), aflatoxin G1 (AFG1), and aflatoxin G2 (AFG2)] are the best known and the most studied among more than 18 different types and metabolites presently identified.

This work aims to present an up-to-date review on the structural diversity, the toxicity, ecological parameters for the production, and occurrence in foods and feeds of as many as possible aflatoxins or their metabolites whenever relevant data are available. However, special emphasis was put on aflatoxin B1 (AFB1) as the flagship aflatoxin for being the most toxic and widespread. A review of the recent publications on aflatoxin occurrence in foods in selected SSA and SSA countries known for their highest dietary exposure is also presented.

## 2. Production, Structural Diversity, and Main Toxicological Properties of Aflatoxins

### 2.1. Aflatoxin-Producing Molds: Taxonomical Elements

The production of aflatoxins has been reported in members of three sections of *Aspergillus* genus; section *Flavi* (B- and G-type aflatoxins), section *Ochraceorosei* (aflatoxins B1 and B2), and section *Nidulantes* (formerly Emericella genus; aflatoxin B1) [11]. However, species of section *Flavi* are the most common and potent aflatoxin-producing moulds, with *A. flavus* and *A. parasiticus* being the most frequently encountered in agricultural products because of their widespread distribution in the agricultural environment and their versatility to grow and produce aflatoxins under different ecological conditions [12,13,14]. A recent classification based on a polyphasic approach revealed that 18 species out of 33 of the section *Flavi* are aflatoxigenic and that each of 16 species is able to produce the four major aflatoxins (AFB1, AFB2, AFG1, and AFG2), while the other two species produce either AFB1 alone (*A. togoensis*) or both AFB1 and AFB2 (*A. pseudotamarii*) [14] (Table 1). The latter authors noted that *A. flavus* strains of Korean origin produce G aflatoxins, contrary to the prevailing view that this species strictly produces B aflatoxins [15,16]. In fact, the production of the G aflatoxins by *A. flavus* was reported when these aflatoxins were first discovered [17], but a controversy was raised when G-aflatoxin-producing strains NRRL 2999, 3000, and 3145, originally identified as *A. flavus*, were re-classified as *A. parasiticus* [11,18]. Subsequently, Wicklow and Shotwell [19] confirmed the production of B and G aflatoxins by other strains of *A. flavus*; NRRL strains 3357, 6412, 6554, 6555, and 13003. Yet, the inability of *A. flavus* to produce the G aflatoxins was later reiterated and evidenced by genetic analysis relating indel (short insertions or deletions) mutations in the cypA/norB region in *A. flavus* to the impairment of the expression of genes coding for P450 monooxygenase enzyme required for the biosynthesis of G aflatoxins [20,21,22]. However, it was argued that this mutation does not occur in all strains, and some *A. flavus* strains can still produce B or G aflatoxins depending on the morphotype (S or L) and on the phylogenetic group (I or II) to which they belong. The morphotypes are defined by the size of sclerotia formed by the strains; “S” for small sclerotia (<400 μ in diameter) and “L” for large sclerotia (diameter >400 μ). In this regard, it was admitted that the phylogenetic group I includes both S- and L-morphotype strains which produce only the B aflatoxins, while group II contains only the S-morphotype strains which produce both B and G aflatoxins [23]. However, it was later demonstrated that the phylogenetic group I strains produce both B and G aflatoxins regardless of the morphotype, and that the phylogenetic group II is not restricted to the S-morphotype strains but contains also the “L” morphotype strains [13]. Furthermore, it was demonstrated that some S-trains (S_BG_) produce both B and G aflatoxins, while others (S_B_) produce only B aflatoxins [24]. Recent taxonomy studies using a combination of advanced analytical techniques confirmed that *A. flavus* can indeed produce B and G aflatoxins irrespective of the morphotype [13,14]. Notwithstanding, it is well established that S-morphotype strains are more aflatoxigenic than their L-morphotype counterparts, and they accumulate larger amounts of aflatoxins regardless of the aflatoxin type [13,24,25]. This was explained by the fact that the production of aflatoxins increases as the size of sclerotia decreases during their formation [25]. Indeed, in the low-elevation regions in Kenya where the S-morphotype is predominating (>90%), the concentration of aflatoxin B1 in maize was reported to exceed 1000 µg/kg [21]. This was practically illustrated by the higher incidence of deadly acute aflatoxicosis in these regions compared with those where the S-morphotype strains are less common [26]. 

### 2.2. Physical, Chemical, and Toxicological Properties of Aflatoxins

More than 18 different aflatoxin types are presently known to occur naturally or as a result of carry over phenomenon in feeds and foods (Table 1). There are about 13 types of aflatoxins that are naturally produced by toxigenic moulds, some of which can be metabolised by human, animals, or other microorganisms to generate derivatives that retain toxicity, although usually with a lower potency compared with the parent molecules. AFB1, AFB2, AFG1, and AFG2 are of the most concern to economy and public health due to their high incidence and high toxicities, especially AFB1. Meanwhile, aflatoxin M1 (AFM1) is of special concern to the safety of dairy products because it is usually carried over in milk of lactating animals fed on feed contaminated with aflatoxin B1 in addition to its high toxicity and potential carcinogenicity in humans [2]. However, the other aflatoxins should not be overlooked because of their intrinsic toxicity, which may not be negligible, or because they can readily invert to the most potent AFB1. They can also be intermediates for the biosynthesis of more toxic mycotoxins [27,28,29]. Table 2 summarises physicochemical and toxicological properties of the major aflatoxins. Supplementary Table S1 provides additional data on other known aflatoxins.

### 2.3. Structural Diversity of Aflatoxins

**Figure 1:**
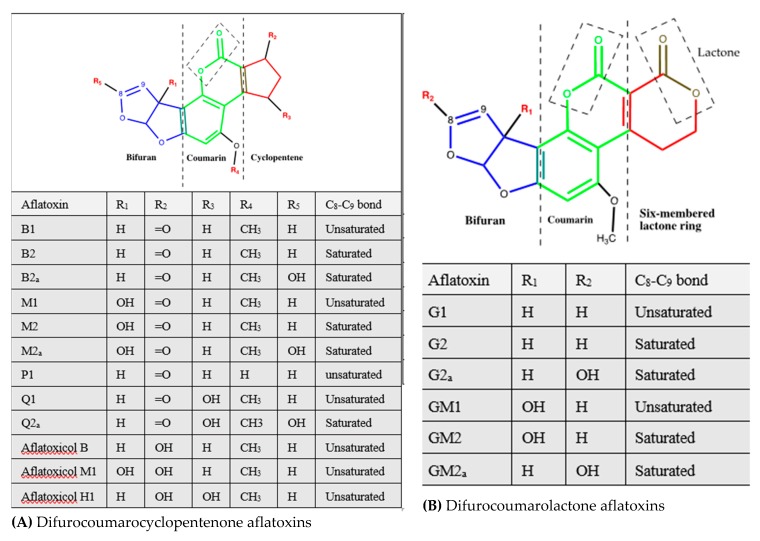

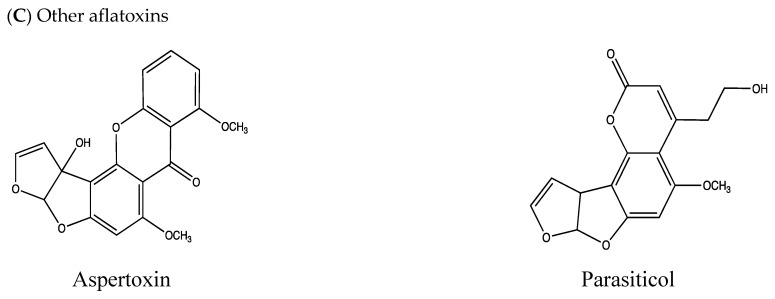
Diversity of chemical structures of aflatoxins in the difurocoumarocyclopentenone (**A**) and the difurocoumarolactone (**B**) groups. Aspertoxin, a difuranoxanthane, and parasiticol, lacking the lactone ring of its parent aflatoxin G1, are occasionally considered as standalone mycotoxins (**C**).

Structurally, aflatoxins are difuranocournarins/difurocoumarins synthesized via the polyketide pathway, and they consist of a coumarin nucleus (Figure 1A,B, in green in the middle) to which are attached a difuran moiety in one side (Figure 1A, left in blue) and either a pentene ring (Figure 1A, in red on the left) or a six-membered lactone ring in the other side (Figure 1B, red on the right). On this basis, aflatoxins fall into two main groups: (i) difurocoumarocyclopentenones comprised typically of aflatoxin B series and derivatives (Table 1 and Figure 1A), and (ii) difurocoumarolactones with the aflatoxin G series as the main representatives, typically including AFG1, AFG2, AFGM1, AFGM2, and AFG2_a_ (Table 1 and Figure 1B). Parasiticol (also designated as aflatoxin B3) is usually considered as a member of the latter group despite the lack of the characteristic six-membered lactone ring (Figure 1C, right) [54]. There also is a question as to whether or not aspertoxin is an aflatoxin due to its bifuroxhanthone structure that does not relate to members of either one of the difurocoumarin groups (Figure 1C, left). This mycotoxin, which is structurally related to sterigmatocystin (an intermediate metabolite of aflatoxins B1 and G1) [28] can also be a precursor of aflatoxin GM1 [38], which may explain the raison for some authors to consider it as a member of the difurocoumarolactone group [63]. Contrary to other aflatoxins, aspertoxin has received the least attention despite its demonstrated toxicity in chicken embryos where it causes malformations, generalized oedema, loss of muscle tone, and haemorrhage from the umbilical vessels leading to death [64]. It is worth mentioning that aflatoxins with saturated (AFG2, AFGM2, and AFM2) or hydrated (AFB2_a_, AFG2_a_, AFM2_a_, AFQ2_a_, AFG2_a_, AFGM2_a_) terminal furan ring are the least toxic, indicating the crucial role that the C^8^ = C^9^ double bond of this furan moiety plays in the toxicity of aflatoxins [45].

## 3. Aflatoxin Production and Incidence in Crops and Feeds

### 3.1. Crop Contamination

As discussed above, aflatoxin-producing moulds, particularly *A. flavus* and *A. parasiticus*, are frequent contaminants of crops where they grow and excrete aflatoxins, which can in turn be found in foods and feeds at high levels making them unfit for consumption. The food and drug administration of the USA (USFDA)considered unavoidable the contamination of agricultural products with aflatoxins that can, at best, be kept at the lowest practical levels to minimize the exposure of humans and animals [65]. However, despite the widespread of aflatoxins throughout the world, their prevalence in foods and feeds is higher in some regions than in others depending on the pedoclimatic conditions, the agricultural practices, the cultivars grown, the mechanical and insect damage of crops, and the awareness of the harmful effects of food-borne toxins on the productivity and safety of produce [66,67]. In addition to inherent traits that influence the toxigenicity of moulds, such as the species, the strain, the morphotype, and the competitiveness within the microbiome [13,23,68,69,70], the level of development of the country; the availability and degree of enforcement of pertaining regulations also account for the extent of food contamination with aflatoxins [71,72]. Considering these factors, the highest incidence has classically been recorded in SSA and SEA countries, owing primarily to the favourable climatic conditions, and then to the low development status and the lack of public awareness of the risk these toxins pose to human and animal health. Table 3 presents the mean annual temperatures and rainfalls in selected countries among the most notorious for the high incidence of aflatoxins in their foods and feeds. Although in each of these countries there are different agro-ecological zones (AEZ) according to the definition of Köppen and Geiger [73], the hot, humid tropical and subtropical climates are predominating and provide ideal conditions for aflatoxin contaminations [67]. The mean annual temperatures in these countries vary between 22 and 29 °C and the mean annual rainfalls are generally higher than 700 mm. Under such conditions, aflatoxigenic molds grow well and produce significant amounts of aflatoxins, especially when the water activity (a_w_) of the produce falls within the range of 0.90 to 0.99 (Table 4). This may be the case if the crop is harvested before its moisture content is low enough (<15%) or stored in an environment with high relative humidity (RH) and poor aeration [24,67,74]. Other growth parameters, such as the pH and nature of the soil, the availability of carbohydrates, nitrogen, phosphates, zinc, and various trace metals also affect the production of aflatoxins [75], but none of which appears to be a limiting factor in the countries considered. These favourable environmental conditions are enhanced by the vulnerability of the prevailing agricultural systems. Farming activities are essentially managed for subsistence by smallholders facing technical and socio-economic challenges that hamper any efforts to restrain aflatoxin contamination [76,77,78]. Moreover, the staple crops grown, such as peanut, maize, sorghum, rice, sunflower, and cottonseed are good substrates for aflatoxin production [79,80].

Since the late 1970s, intensive research has been conducted to assess the extent of aflatoxin contamination of different foods and feeds in these regions, and the results were used by the IARC working groups to relate aflatoxin dietary intake to liver cancer. Different analytical techniques have been used to generate quantitative data on aflatoxins in crops. These include the classical techniques, such as thin layer chromatography (TLC), enzyme-linked immunosorbent assay (ELISA), high performance liquid chromatography (HPLC), and more recently the advanced and more sensitive techniques and reliable such as liquid chromatography tandem mass spectroscopy (LCMS/MS) are being increasingly used when affordable by research or control laboratories. Published data on the contamination of staple crops with aflatoxins in selected countries from SSA (West and South-East regions) and South-Eastern Asia are summarised in Table 5 and Table 6 with more extensive data in supplementary Tables S2 and S3, respectively. The tables show that peanut/groundnut and maize are the most highly and frequently contaminated crops, whereas millet and rice are generally less contaminated, although not always with safe levels. For example, despite the generally low AFB1 contamination of rice in three provinces of Vietnam (Table 6 and Supplementary Table S3), risk assessment associated with AFB1 in rice was higher than that recorded in other Asian countries (Thailand, Japan and Hing-Kong), and was particularly high for the population of Ha Giang province (21 cases of liver cancer per 100,000 people and per year in adults) [81]. This was related to the high average consumption of rice in Vietnam, which ranges, for adults, between 244.4 and 300.5 g/day [81]. The climates that predominate in AEZs where the highest aflatoxin levels were recorded are warm arid and semi-arid, tropical, or subtropical or irrigated desert [75] (see also, Table 5 and Table 6; and Supplementary Materials in Tables S2 and S3 for details). In addition to the climate type, an annual mean rainfall around 700 mm is an additional factor that favours aflatoxin contamination [24]. A positive relationship between the rainfall and aflatoxin concentration was demonstrated in sorghum grown in four different AEZs in Nigeria, where the contamination with aflatoxin B1 was highest in the zone with rainfalls exceeding 1400 mm [82]. Nonetheless, aflatoxin contamination of peanut and maize was reported to be maximal at an average annual rainfall between 600 mm and 700 mm and decrease exponentially thereafter [24]. This may partly explain the consistently high aflatoxin levels and incidence in foods (Table 5) in Kenya reaching where the mean annual rainfall is about 670 mm (Table 4). In this country, the aflatoxin incidence in maize varied between 25% and 98% with contamination levels reaching 48.000 mg/kg (Table 5 and Table S1). According to a survey on aflatoxin contamination of maize conducted in the country during the period 2006–2009, only 17% of the production is fit for human consumption [83]. However, due to food shortage and lack of awareness of the inherent health risks, in addition to the absence of official control, these foods are eaten by local populations, which explains why this country has been repeatedly and severely afflicted by aflatoxicosis outbreaks [84,85]. Recent data suggest that that the situation did not improve since then and the dietary exposure to aflatoxins remains too high. The probable daily intake (PDI) of aflatoxin B1 in the country, via maize only, was recently estimated to vary between 0.07–60,612.00 ng/kg bw/day, with an average of 312.4 ng/kg bw/day) [86], which is alarming compared with an average of 10 to 200 ng/kg/day for the rest of the world [87], and with the conservative tolerable daily intake (TDI) of 0.11 to 0.19 ng/kg bw/day [88]. Incidentally, this country also ranks among the countries with the highest prevalence of oesophageal cancer, which was associated with aflatoxin intake as a risk factor [21,86,89]. Conversely, a recent survey on aflatoxin contamination of maize grown in eight different AEZs in Uganda revealed that the highest levels ( a maximum of 3760 µg/kg and an average of 66.5 μg/kg were recorded in the zones with high rainfalls (>1200-mm); the percentage of samples exceeding the national regulatory standards of 10 μg/kg reached 22.2% [90].

It should be pointed out, however, that despite the well-established impact of the climate type on the extent of crop contamination with aflatoxins, no direct correlation between aflatoxin levels and the AEZs has been established. On the contrary, in a survey on aflatoxin contamination of maize and groundnut samples collected from 27 districts of three different AEZs in Zambia no such correlation could be established [24]. The levels of aflatoxins in a product within the same AEZ were shown to vary greatly depending on the rainfall and temperature variations from one year to another, in addition to the experimental design and the sampling point [91]. In fact, there is a multiplicity of factors that interfere with the effect of the climate type at different production stages from plant development to crop storage [21,67]. In SSA, there is a broad spectrum of AEZ spanning from highly humid costal savanna and rainforest to the arid savanna in the North along the desert belt of Sahel. The humid hot climates of the tropics are more favourable to aflatoxin production than the temperate, arid or cool climates [92]. However, aflatoxin contamination of crops grown in the region does not necessarily follow this climatic distribution. For example, despite the favourable ecological conditions for aflatoxin production of the humid tropics along the Western cost of the Atlantic sea, maize and groundnut were shown to be inconsistently contaminated depending on the country; and very low contaminations were reported in zones assumed to be at high risk [93,94,95,96]. Conversely, in the sub-desert savanna of Sudan assumed to be at low risk owing to low rainfall for a short period (July to September), crops are highly contaminated due to the frequent drought stress and the traditional field-drying and storage leading to poor quality and insect damaged crops [97]. The impact of AEZ climate types and other interfering factors on aflatoxin crops contamination in different regions of SSA were thoroughly reviewed by Cardwell and Henry [92]. In fact, it is now well-recognized that management systems with rigorous implementation of the good agricultural practices (GAPs) and farmer trainings are critical measures to mitigate the incidence of aflatoxins in agricultural products regardless of the climate type [21,72,86,98,99,100].

The implementation of such measures in South Africa in the framework of a project on the adaptation of agricultural practices to climate change in SSA within the “comprehensive Africa agriculture development programme (CAADP)” aimed at “good agricultural adaptation practices” [112], appears to have been successful in controlling aflatoxin contamination. The most recent survey in the country revealed very low incidence and contamination levels of AFB1 in peanut and wheat samples collected from all the country regions during the period of 2014 and 2018 [113]. Nonetheless, climatic shifts and occurrence of drought periods followed by heavy rains that occur in the region, remain a challenging issue which may counteract these measures and induce a rebound in the levels of aflatoxin contamination [67,114]. Indeed, the major documented aflatoxicosis outbreaks were reported to coincide with drought periods followed by unseasonal heavy rain or in regions with frequent and unpredictable temperature and rainfall shifts due to the so-called El Niño-Southern Oscillation (ENSO) phenomenon [115]. A first outbreak in India in 1974 was caused by the consumption of contaminated maize in two chronically drought-stricken districts which received unseasonal rain while the maize was mature and ready to harvest [116]. In Kenya, the major aflatoxicosis outbreak of the year 2004 was preceded by a severe drought followed by heavy rains during the harvest period of the maize implicated in the outbreak [84], as discussed below (Section 4.2 and Table 5). The same country has experienced another drought in 2009, which was also followed by a significant increase in maize contamination with aflatoxins resulting in the condemnation of 10% of the production in the following year [117,118]. The water stress caused by drought weakens the plant defence and increases its susceptibility to mould infection and aflatoxin production [91], which may be further enhanced if the crop is harvest in the rainy season. A recent survey on the contamination of food products of West Africa SSA countries revealed that aflatoxin contamination of crop samples collected during the rainy season was significantly higher than those collected during the dry season [80]. Similar observation could be made from a comprehensive survey on aflatoxin contamination of various foods in Thailand for the period of 1969–1970 [119,120] (see also Table 6). This highlights the primary impact of temporal distribution of rainfall, rather than its quantity throughout the year, on the extent of aflatoxin contamination. Harvesting during the rainy season yields crops that have not yet reached low-enough moisture content to resist mould colonization. Yet, moisture content of the crop at harvest is not the only explanation of the phenomenon, as aflatoxin contamination was shown to be highest when high rainfall occurred during the pre-flowering stage and lowest during the flowering and post-flowering stages, not including harvest [69]. This was explained by the healthy status of the plant in the latter stages, which should be accompanied by a high vegetation cover, which increases the plant’s resistance to mould invasion and aflatoxin production [69].

Inappropriate storage conditions also play a major role in the increase of aflatoxin contamination; and increased aflatoxin levels from field to storage structures is well documented. For example, a 26-fold increase in aflatoxin concentration was observed in sorghum grown in Niger state (Nigeria) from field to storage in traditional mud-built barns [82]. Also, the maximum aflatoxin concentration in maize increased from 26.5 μg/kg at harvest to 1460 μg/kg after 4 months of storage at the farmers’ household in South-eastern Nigeria [121]. Moreover, Villers [117] quoted that aflatoxin concentration increased by 200 times in peanut after 2 months of storage under conventional conditions in Mali and by 300 times in maize after 3 months of storage in traditional facilities in Uganda. Such results were corroborated on bambara nut (*Vigna subterranean*, L), groundnut, maize, sunflower, and sorghum from different AEZs in Tanzania [100]. Strikingly high aflatoxin levels were recorded in commercial peanut samples collected from different marketing structures in Kenya, with the highest levels, e.g., 32,328 μg/kg, being recorded in informal market outlets and poorly designed stores of retailers and stockists [122], see also Table 7 and Table 8. Under experimental conditions, aflatoxin concentrations in both peanut and maize increased by more than 1000 times after one week of storage at 31 °C and 100% relative humidity compared with safe levels in freshly harvested crops [24]. BIOMIN research centre (Tulln, Austria) has recently conducted a worldwide comprehensive survey on food and feed contamination with various mycotoxins emphasising the continued leading position of SEA and SSA in aflatoxin contamination of foods and feeds [123].

The study revealed that 76% and 57.4% of the analysed samples were contaminated with median concentrations of 23 and 10 μg/kg, respectively. These values were significantly higher than those recorded in the other regions of the world. The levels of AFB1 exceeded the highest maximum tolerable level (MTL) guidance of 20 μg/kg in 38.5% of the samples collected from SSA and 20.9% of those from SEA [123]. The percentage of the samples containing AFB1 concentrations exceeding the highest MTL varied between 0.0 and 6.6%, with the exception of the East Asia (41.1%), which is another high-risk region with climatic and socio-economic conditions similar to SEA. Moreover, the same study reported that co-occurrence of AFB1 with each of deoxynivalenol, zearalenone, fumonisins or ochratoxin in maize is rather common, and was detected in 14, 14, 22, 12% of samples, respectively.

The situation of crop contamination with aflatoxins that prevails in SEA region is similar to that describe above for the African countries, with maize being the most highly and frequently contaminated in all of the countries listed in Table 6. Despite the vast area covered by the region, its climate types are less diversified than in SSA countries. The climate in most countries of the region is mainly tropical or subtropical with narrow mean annual temperature variations (21 °C and 29 °C), and a high annual rainfall (Table 4). The region is subject to monsoonal weather system producing marked rainy and dry seasons during the year, thereby providing favourable conditions for mould growth and aflatoxin production [167]. The predominating climate sub-types in the AEZs with high levels of aflatoxin contamination are Aw (*tropical savanna*), Cwa (*humid subtropical), and Af* (tropical rainforest) (Table 6 and Table S3) Among these countries, India, Malaysia, and Thailand have experienced episodes of aflatoxicosis traced to the consumption of heavily contaminated foods with aflatoxins [116,168,169], consistent with high aflatoxin levels recorded in their staple crops, including peanut, maize, and sorghum. In contrast, rice that represents the primary staple food in this region, is the least contaminated according to the published data reviewed in this study (Table 6 and Table S3)

### 3.2. Feed Contamination

Crop residues and by-products from grain mills and/or oil extraction factories are often used as animal feeds. In developing countries, mouldy cereals and nuts of low grades are generally sorted to be fed to animals either directly or as ingredients in manufactured feeds [82,138,170]. Therefore, it is reasonable to anticipate that such feeds are more likely to be highly contaminated with aflatoxins than their counterpart crops destined to human consumption. This appears to be valid in most SSA, especially in Kenya and Nigeria where aflatoxin concentrations in feeds are particularly high [83,171]. A comprehensive survey on aflatoxin contamination of feeds and feed ingredients in Asia-Oceanian countries, including Malaysia, Philippines, Thailand, Indonesia, and India (SEA) showed that 30.3% of the samples contained AFB1 at an average level of 46.0 μg/kg and a maximum level of 4278.0 μg/kg [172]. Moreover, the levels of aflatoxins in commercial poultry feeds were demonstrated to be significantly higher than the maize used as ingredient in their formulation [121,173]. Nevertheless, feed contamination with aflatoxins may not necessary correlate with that of ingredients used in feed formulations, depending on the type and composition of the feed, the processing steps when applicable, and considerations of quality grading. For instance, maize for feed manufacture in Indonesia was separated into three grades of decreasing quality before being analysed for aflatoxin contents. The quality of the maize was determined visually on the basis of the proportions of foreign materials and mouldy, dead, or damaged kernels, as per the Indonesian grading system routinely practiced by the feed milling industry [174]. Unexpectedly, the results showed that aflatoxin concentrations increased from the best to the worst grade of the grains, suggesting that the grading system relying on visual inspection does not reflect *a priori* extent of contamination. The relatively high aflatoxin levels recorded in feeds of the regions are of concern to both animal and human health, since they are not only detrimental to livestock but can also be carried-over to human via foods derived from these animals, such as eggs, meat, and milk [175].

## 4. Toxicity of Aflatoxins and Major Aflatoxicosis Outbreaks

The toxicity of aflatoxins to humans and animals through food and feed consumption and their association with acute and chronic diseases is well established [87]. However, the degree of toxicity and the toxicological effects vary greatly depending mainly on the aflatoxin type and the host. AFB1 is, by far, the most toxic aflatoxin, followed by AFG1, AFB2, and AFG2, while AFM1 has a similar toxicity as AFG1 [176,177]. The other less toxic aflatoxins and those considered to be “non-toxic” or detoxified forms are still of concern to public health due to their inherent, although weak, toxicities with potencies ranging between 0.1% and 50% compared with AFB1 [45] (see also, Table 2). Most importantly, they can invert to their highly toxic precursors in foods or after ingestion [30]. For example, aflatoxicol, which is 25 to 50% as potent as its parent AFB1, is almost entirely converted back in the liver to either the more toxic parent AFB1 or to AFM1 [1,178,179].

### 4.1. Aflatoxicoses

The ingestion of aflatoxins at high levels in a single dose or repeatedly for a short period of time induces acute intoxication, hereafter designated aflatoxicosis, in humans and animals with typical symptoms, including jaundice, lethargy, nausea, edema, hemorrhagic necrosis of liver tissues, bile duct hyperplasia, and eventually death (10–60%) subsequent to severe liver damage [180]. Although there is no consensus on the specific dose of aflatoxins that triggers acute toxicity in humans, it is well established that such a dose is highly variable depending on many factors, including the age, gender, health and nutritional status, presence or absence of underlying factors (e.g., chronic viral hepatitis, alcoholism, smoking, cirrhosis, exposure to hepatotoxic microcystins); and it is lowest in youngsters, as substantiated by the highest death rates of this age-group in aflatoxicosis outbreaks [181,182,183]. A rough estimation of the acute dose of AFB1 concerned a case report on a-15-years old Ugandan child weighing 36 kg who has been eating AFB1-contaminated cassava on a daily basis until he died by liver failure [184]. The authors calculated the likely cumulative amount of cassava that caused the child’s death to be 3.1 kg contaminated with 1.7 mg/kg, corresponding to a total dose of 146 μg/kg bw that he had eaten in 22 successive days before death. However, these calculations are very approximate, as they were based on the lethal dose of AFB1 to monkeys, and on the assumption that the AFB1 concentration in cassava, determined retrospectively after the death, was constant throughout the whole period of intake preceding the death. Nevertheless, the outcome of these calculations, is in accordance with an estimation of the world health organization (WHO) based on records of aflatoxicosis outbreaks worldwide and in vitro tests, which considers that regular consumption of food contaminated with 1 mg AFB1/kg or higher for a short period causes acute intoxication in humans [177]. According to the same report, daily consumption of food contaminated with AFB1 at a dose of 0.02–0.12 mg/kg bw over 1 to 3 weeks causes a life threatening aflatoxicosis. Furthermore, the cumulative lethal dose in humans was suggested to vary from 10 to 20 mg for adults and 3 mg for children [185], which is also consistent with the estimated total dose of ~5.3 mg ingested in 22 days by the Ugandan teenager [184]. Nonetheless, deliberate ingestion of 5.5 mg chemically pure AFB1 over two days and 35 mg over two weeks in suicide attempts by an adult women from the USA was reported to cause no serious aflatoxin-related injuries at admission to the hospital for mild symptoms and even 14 years later [186]. Although difficult to explain, this could be due to her overall well-balanced nutritional status, age, and gender, since well-nourished adult females are less susceptible to aflatoxins than males of similar health and nutritional status [187,188].

In animals, the lethal dose varies greatly among species, as suggested by the wide variation in their LD_50_ values ranging between 0.3 and 18.0 mg/kg bw [189], although values as low as 0.2 mg/kg bw or as high as 60 mg/kg bw were occasionally reported (Table 2). Animals like ducks, sheep, turkeys, dogs, pigs, and rats are the most susceptible, whereas monkeys, chickens, mice, and ruminants the most resistant [168,190]. The higher susceptibility of the first group of animals was explained by their ability to metabolize rapidly AFB1 via the phase II metabolism driving towards the formation of aflatoxin-albumin adducts [191]. In a study on the impact of an orally administered single dose of AFB1 to mice, 0.66 mg/kg bw induced severe tissue injury 5 days after the ingestion [190]. In poultry, the AFB1 doses that killed all tested birds varied between 0.8 and 4.0 μmg per animal, with turkeys being the most sensitive (0.8 mg) and geese the most resistant (4.0 mg), whereas no death was observed in chickens at the highest doze of 4.0 mg [189].

With the exceptions of the Ugandan child and the US laboratory worker, no specific doses to our knowledge, have been reported on a given individual situations that may inform on the minimum levels of aflatoxins that induce a response in humans. Although, the outbreak intoxications discussed below (Section 4.2) provide valuable epidemiological information, they do not allow accurate calculations of the daily intake individually before the onset of the intoxication to establish a dose-response relationship. In all these cases, the implicated food is analysed retrospectively, and its contamination is a way beyond the legal maximum tolerable limits.

### 4.2. Major Aflatoxicosis Outbreaks

Although aflatoxins are most known for chronic diseases, mainly liver cancer, their association with acute intoxications is well established, and acute aflatoxicosis outbreaks making many victims have been reported in endemic countries in SEA and SSA Table 7 summarizes the main of such outbreaks that have been documented in Asia and Africa, and their circumstances. No aflatoxicosis cases or outbreaks has been reported, to our knowledge, in industrialized countries due to the low exposure which is 100-fold lower than that recorded in developing African and Asian countries (1 ng/kg bw per day vs. 100 ng/kg bw per day) [177]. The first significant aflatoxicosis outbreak occurred in two Indian regions encompassing more than 200 poor setting ethnic villages with a protein deficient nutritional status who relied mainly on maize as a food source. The climate in these neighbouring Western Indian regions is typically hot desert (BWh) and hot semi-arid (BSh) characterized by low annual rainfall and chronic drought. In 1974, these regions received abundant unseasonal rain (October–November instead of the usual rainfall period of June–September), while the maize standing in the field had attained the full maturity stage to be harvested [116]. Shortly after that, an epidemic struck affecting people in family clusters and the pets sharing the same diet. The possibility of an infectious disease was ruled out, as it was not contagious and the prescription of anti-microbial drugs prove ineffective [192]. Clinical and post-mortem histopathological examinations of dead victims revealed obvious symptoms and liver lesions evoking aflatoxicosis; i.e., periportal hepatic fibrosis, and bile duct proliferation. Thin layer chromatography (TLC) analysis showed the presence of unidentified green and blue spots in extracts of necropsy liver samples and AFB1 in the serum of some patients. Moreover, the suspect maize was highly contaminated with *A. flavus* and contained aflatoxins at concentrations ranging between 6500 and 15,600 μg/kg. Exposure calculations suggested that the populations have been ingesting, through their diet, 2–6 mg of aflatoxins on a daily basis for several weeks from the start of harvest to the depletion of maize stock, which coincided with the end of the outbreak [116]. Together, these data were taken for an evidence to ascribe the epidemic to a maize-born aflatoxicosis (Table 7).

In the SSA region, Kenya has been the most severely afflicted by aflatoxicoses, especially in the East-central region where the prevailing climate is hot semi-arid (BSh), humid subtropical (Cwa), or oceanic tropical highland (Cwb) with frequent alternation of dry and rainy periods. Populations of these regions, mainly of the Akamba/Kamba tribe, grow maize for home consumption as the main staple food and store it by traditional means in containers that they place inside a granary or hung to the ceiling of the kitchen [85]. Two notable aflatoxicosis outbreaks were recorded in the same region of the country (Table 7). The first one occurred in 1981 after a severe shortage in rainfalls during the year 1980 followed by heavy rainy season that extended from October to May instead the normal period of October to December (short rainy season). Starting from late March to early June 1981, 20 patients, mostly from two family groups of Makueni district, were admitted to the provincial hospital with jaundice and other symptoms suspecting a viral hepatitis [85]. Within 22 days of the early symptom onset (i.e., abdominal discomfort, anorexia, general malaise, and low-grade fever), 12 of the patients developed massive ascites and gastrointestinal haemorrhage before they died from liver failure. Among those, six were from the same family of eight members including two twins who were not affected, as they were not fed the family diet.

From another family of seven members, four had the illness and two of them died, while the other two recovered progressively within 20 days of hospitalization. Both families were fed on inadequately stored maize as per the traditional Akamba method described above and in a wet environment [85]. In each of these families, the stored maize was found to be contaminated with AFB1 at concentrations of 12,000 and 3200 μg/kg, and AFB2 at concentrations of 1600 and 2700. As was the case in the Indian outbreak, the onset of the disease in the family members followed immediately the death of dogs sharing their diet. After necropsy, AFB1 was detected in liver samples of two deceased children at levels of 39 and 89 μg/kg, which was considered as an additional evidence supporting the causal effect of aflatoxins. Two other fatal cases tested positive for HB virus surface antigen (HBsAg), suggesting a pre-existing liver damage that increased the susceptibility to aflatoxins of the patients. Continuous dietary intake of sublethal or subclinical doses of aflatoxins in addition to protein deficiency of the diet, due to the food shortage in the previous year, were suggested to have contributed to the increased susceptibility of the victims [85].

The second episode of aflatoxicosis outbreak that occurred in Kenya in 2004 was the most significant worldwide, as it caused 317 cases with 125 deaths (Table 7). It also covered a larger zone encompassing four districts of more than 40,000 km^2^ populated by 2.8 million inhabitants, mostly of the Akamba tribe. Makueni and Kitui districts were the most severely affected (47% and 32% of cases, respectively), followed consecutively by Machakos and Thika with 6% and 4% of the total cases. In an almost identical scenario as for the former aflatoxicosis outbreaks in India (1975) and Kenya (1981), this one also occurred after unseasonal heavy rain preceded by a year of severe shortage in rain and foods, which resulted in high aflatoxin contamination of maize and increased susceptibility of nutritionally deficient rural farmers [84]. During the course of the aflatoxicosis, a survey was conducted in June 2004 in the households and market outlets to assess the aflatoxin contamination of home-grown and market maize. The highest contamination levels were recorded in samples collected from home-grown maize stored in households as compared to those of the maize sold in market outlets in the geographic area of the outbreak. In households with victims, the maize was stored under damp conditions and 48.4% of the samples contained between 20 and 8000 μg/kg of aflatoxins [193]. These considerations and the absence of viral agents, as demonstrated by serological tests in a case-control study, led the investigators to relate the aflatoxicosis to the home-grown rather than the market maize [84,193]. Yet, the contribution of market maize as a continuous source of aflatoxin intake outside the season or when the stock of the household maize is exhausted was highlighted by the authors. Overall, aflatoxin concentrations exceeded the Kenyan maximum tolerable limit (MTL) of 20 μg/kg in 55% of the analysed samples collected from market maize, and the levels of contamination in samples from each of the affected districts were consistent with the number of reported cases. The highest geometric means of aflatoxin concentrations in maize, 52.91 μg/kg and 35.27 μg/kg, were recorded in Makueni and Kitui, respectively. Conversely, maize samples collected from Machakos and Thika markets were the least contaminated, with geometric means of 17.84 and 7.52 μg/kg, respectively. This reflects also the aflatoxicosis ratios per 100,000 inhabitants in these districts. In Makueni and Kitui, the aflatoxicosis ratios varied from 34.8 to 77.5 in the northern areas and from 12.6 to 34.7 in the southern area, whereas they were much lower (0.66 to 12.5) in both Machakos and Thika [84]. Nonetheless, in Thika, the least affected district, the maximum aflatoxin concentration was as high as 46,400 μg/kg, which is sufficient to trigger severe aflatoxicosis after one or few servings in susceptible persons [177]. Of the total analysed samples, 7% were contaminated with more than 1000 μg/kg, whereas at the district level, samples containing such high concentrations represented 12%, 10%, 3%, and 4% of the samples collected from Makueni, Kitui, Machakos, and Thika markets, respectively [84]. In addition to the results of the survey indicating the exceptionally high contamination of the maize locally produced and consumed in these Kenyan provinces, a case-control study was conducted separately to relate AFB1 dietary intake to the disease by titrating the biomarker AFB1-albumin adduct in serum [195]. The study, conducted on 40 selected case-patients and 80 suitable controls, demonstrated a high correlation between the titre of the adduct in the serum and aflatoxin intake via maize consumption. Moreover, the adduct titre in HBsAg negative case-patients was 22.2 times higher than that of the controls, therefore, clearly establishing the relationship between aflatoxin intake and the disease. Later, another study focused on the identification of mould species contaminating the maize responsible for the outbreak showed that out of 1232 mould strains isolated from home-grown maize and inadequately stored at the households with victims, 97.8% and 2.1% were identified as *A. flavus* and *A. parasiticus*, respectively [196]. Isolates of *A. flavus* were largely predominated by the S-morphotype representing 71.8% of the isolates, compared with 28.2% of the L-morphotype. The study also showed that the incidence of the S-morphotype was highly correlated with the concentration of aflatoxins B in the maize, and strains of this morphotype were the exclusive contaminants of 5 samples out of 6 containing more than 1000 μg/kg of aflatoxins. Conversely, *A. parasiticus* was weakly represented (28.2% of the total isolates) and only detected in samples with aflatoxin contents lower than 260 μg/kg [196].

In late 1988, during the 9 days of the nine-emperor-gods festival held in Malaysia [197], the consumption of a traditional Chinese dish called Loh See Fun was implicated in a poisoning outbreak resulting in 17 severe cases and 13 deaths. Patients were children of 2.5 to 11 years of age, with one case of a 46-years-old man, and only children died. Other patients (45 in number) who ate the same dish as the affected cases, developed similar but milder and transient symptoms; they were, hence, considered as presumptive cases and discarded from further investigation [182]. The main ingredient of the offending dish consisted of white noodle made with a mixture of rice and corn flour. Boric acid was illicitly added to the dish by the producing factory in Kampar city (Malaysia) to extend its shelf life and enhance its sensory properties [185]. The onset of the poisoning was fairly rapid and the first symptoms evoking a Reye-like syndrome appeared within a mean time of 8.5 h after the ingestion. The patients exhibited different symptoms, the commonly observed of which were vomiting, seizure, diarrhoea, abdominal pain, anorexia, and coma. Jaundice was generally weak at the beginning and increased in severity with time until the eventual death with liver and kidney failure. Depending on the patient, the survival time varied from 2 to 9 days with a mean of 5 days [182]. The results of clinical, analytical, and histopathological examinations ascribed the intoxication to both boric acid and aflatoxins. The boric acid poisoning (BAP) was mainly indicated by metabolic acidosis, acute renal failure, and the relatively short survival time. On the other hand, aflatoxicosis was indicated by the initial symptoms evoking Reye-like syndrome followed by liver injury and failure with bile duct proliferation, as the health status deteriorated leading to death. However, the detection of abnormally high levels of aflatoxins B1, B2, G1, M1, and M2, and aflatoxicol in various organs, including liver, kidney, heart, spleen, lung, and brain was the main supportive feature of the aflatoxicosis, although it does not exclude BAP [182]. Boric acid and aflatoxins may have acted synergistically, as indicated by diagnostic features that characterize one or the other disease but not both.

A recent aflatoxicosis outbreak was reported in the central region of Tanzania in 2016 [183]. The prevailing climate in the region is hot semi-arid (BSh) and subject to frequent alternations of drought and flood periods caused by ENSO [115]. This phenomenon induces extreme shifts in rainfall and temperature causing both severe drought and rainfall events, usually followed by increased incidence of disease outbreaks, as it creates favourable ecological conditions for microbial pathogens and their vectors to emerge. According to the latter study [115], a strong El Niño hit Tanzania in 2015–2016 and raised above normal the cases of malaria and cholera in the period of April 2015 to March 2016, which continued through 2017 for cholera. Although not mentioned in the study, this situation applies to the aflatoxicosis outbreak that occurred in the period of 14 May to 14 November 2016 (Table 7). The outbreak affected 68 individuals in family clusters and killed 20 of them. Before death, the patients presented typical symptoms of aflatoxicosis, i.e., jaundice, abdominal pain, vomiting, diarrhoea, and ascites. A house-to-house survey conducted in selected households including case-households with victims and those without (controls), showed that more than 50% of the cases were children below 15 years-old who had eaten home-grown maize contaminated with both aflatoxins and fumonisins at abnormally high levels [183]. Aflatoxin contamination in samples collected from case-households was significantly higher than those of controls (10–51,100 μg/kg versus 2.4–285 μg/kg). Fumonisins were detected in the maize sampled from case-households at concentrations ranging from 945 to 12,630 μg/kg. Of the maize samples contaminated with both mycotoxins, 80% exceeded the regulatory standards of 10 μg/kg and 2000 μg/kg for total aflatoxins and fumonisins, respectively. Moreover, the titres of aflatoxin-albumin adduct in the serum of case-patients usually exceeded 1000 pg/mg and were 3.6 to 8.2 times higher than in the serum of controls (36–32,800 pg/mg vs. 10–4020 pg/mg) [183]. The increase in aflatoxin-albumin adduct titre is a strong indication of the causal link between aflatoxins and the outbreak, the severity of which may have been increased by an additive effect of fumonisins [198].

According to the magazine “Outbreak News Today”, during the period of 20 June to 13 July 2017, two clusters of eight children from two different villages of Kiteto District (Manyara region), North Tanzania, were admitted to the hospital for suspicion of aflatoxicosis [194]. They were presenting the common symptoms of acute aflatoxicosis, namely general malaise, loss of appetite, vomiting, abdominal distension and pain, dark stools without diarrhoea, and jaundice. Three cases of the first cluster, consisting of five children (three to nine years-old), died shortly after the admission. The 3-years-old child died after two days and the other four were transferred to the regional referral hospital of Dodoma for more intensive care; and two of them died two days later. On 13 July 2017, the three children (one of four-years-old, and two of ten years-old) of the second family cluster were admitted to the hospital with similar symptoms as the previous patients plus an altered mental status [194]. The four-years-old child died within hours after admission, meanwhile the other patients were referred to the regional hospital with the two survivors of the first cluster. As per the date of the report (24 July 2017) the four survivors were still hospitalized and there has been no update on the situation to our knowledge. All the children were reported to have consumed improperly stored maize, which in conjunction with the symptoms suggests that the disease is likely to be an aflatoxicosis [199].

In addition to the above-mentioned aflatoxicosis outbreaks, some sporadic cases have contributed to increase the scientific knowledge on the toxicity of aflatoxins. For example, the death of a Ugandan teenager in 1967 who had been fed regularly on mouldy cassava as a staple meal and the association of his death with aflatoxicosis, as evidenced by the typical liver lesions observed upon post-mortem histopathological examination and the high contamination of the cassava meal (1700 μg/kg), was the first demonstration of the acute toxicity of aflatoxins in humans [184]. Also, intentional ingestion by a 25-years-old laboratory female worker who attempted to commit suicide of 5.5 mg of pure AFB1 over two days and another dose of 35 mg, six months later, over two weeks and developed only minor transient symptoms (rash, nausea, and headache) gave some insights on the difference in susceptibility to aflatoxins among individuals [186].

## 5. Risk Assessment of Aflatoxin Dietary Intake

Considering the well-established adverse health effects of aflatoxins, international and regional organizations have been actively working to provide quantitative estimates of the risk associated with their consumption. SEA and SSA countries were the first target countries for risk assessment studies due to the notoriously high incidence of aflatoxins in the staple crops they produce and for the prevalence of liver cancer among their populations. Therefore, in addition to toxicity data generated from experimental animals, epidemiological studies conducted in these regions were also used to document the earliest quantitative risk assessment on aflatoxins [200]. In this study, the joint Food and Agriculture Organization (FAO)/World Health Organization (WHO) Expert Committee on Food Additives and Contaminants (JECFA) designed a deterministic approach to assess the risk in different countries of world as the multiplication products of the carcinogenic potency (P_cancer_) and the dietary exposure to AFB1 in each country [200]. The P_cancer_ was defined as function of seropositive (HBsAg^+^) and seronegative (HBsAg^−^) individuals for surface antigen hepatitis B virus as follows:

P_cancer_ = (PHBsAg^+^ × FHBsAg^+^) + (PHBsAg^−^ × FHBsAg^−^) (1)

where, P_cancer_ is the average carcinogenic potency of AFB1 expressed as the number of cancers per 100,000 individuals per ng of AFB1 per kg bw per day; PHBsAg^+^ and PHBsAg^−^ as the carcinogenic potency of AFB1 in HBsAg^+^ and HBsAg^−^ individuals, respectively; FHBsAg^+^ and FHBsAg^−^ as the population fractions of HBsAg^+^ and HBsAg^−^, respectively.

In this approach, FHBsAg^+^ was estimated to be 25% for developing countries, including those of SEA and SSA with high incidence of liver cancer and Hb virus chronic infections. Due to uncertainties in the determination of the specific additional risk caused by HB infection [201], the conservative figure of 30 times higher risk in HBsAg^+^ than in HBsAg^−^ individuals was adopted. Accordingly, PHBsAg^+^ and PHBsAg^−^ were estimated to be 0.3 and 0.01 cancers/year/100,000 individuals per ng AFB1 per kg bw per day, respectively. These values were derived from modeling of the results of epidemiological studies conducted in Guangxi (The Peoples’ Republic of China) [202]. Therefore, the average P_cancer_ used to determine the risk for cancer in developing countries was calculated as:

P_cancer_ = 0.3 × 0.25 + 0.01 × 0.75 = 0.083 cancers per 100,000 individuals per ng of AFB1 per kg bw per day, and the ensuing cancer risk for the lifetime exposure (60 years) according to the deterministic approach, was calculated as:

R = 0.083 × EDI(2)

where R is the risk for lifetime exposure expressed as numbers of cancers per year per 100,000 individuals. EDI is the estimated daily intake, or exposure, expressed as ng AFB1 per kg bw per day and calculated by the multiplication of the contamination level (ng/g) by the consumption rate (g/day) of contaminated food per kg body weight.

Taking these parameters and equations into account, the additional risk for primary liver cancer (HCC) determined for African countries was shown to be generally higher than 1 cancer/year/100,000 individuals (Table 8). This is regarded as a risk of concern and usually requires appropriate management measures by the food safety authorities to be reduced. The highest risks for primary liver cancer were associated with maize and a maize-product, kenkey, in Kenya and Ghana, respectively, which emphasizes again the vulnerability of these countries to aflatoxin contamination, especially in the rural areas (Table 8). Although maize did not seem to be a real problem in The Gambia, the risk posed by millet, groundnut and, to some extent, rice in the country was of concern and warranted appropriate management measures if the objective of less than one additional liver cancer per 100,000 individuals were to be met.

The risk for liver cancer associated with dietary intake of aflatoxins, as genotoxic hazards, was also estimated by a different approach developed by the European Food Safety Authority (EFSA) using the benchmark dose (BMD) [203]. According to this approach, the BMD is defined as the dose that gives a low but measurable extra risk of 1 to 10% compared with control; it is calculated by the use of mathematical models for dose-response (risk characterization). The BMD value that corresponds to the lower limit of one-sided 95% confidence interval is designated benchmark dose limit (BMDL), which is further specified as, e.g., BMDL1, BMDL5 or BMDL10 when the extra risk chosen for its determination is 1, 5 or 10%, respectively [203]. The ratio of the chosen value, usually BMDL10, divided by the exposure (EDI) yields the margin of exposure (MOE) which indicates whether or not the risk posed by the hazard should be managed for increased safety. For genotoxic hazards, such as aflatoxins, an MOE of 10,000 or higher indicates that the risk is too low to require specific management measures by national food safety authorities. Applied to the risk associated with the main agri-foods susceptible to AFB1 contamination in African countries, this approach showed that MOE values are generally very low compared with the 10,000 value indicative of no concern (Table 6). The outcomes of both JECFA and EFSA approaches indicated clearly that AFB1 intake in these countries is of high concern to public health and that there was a prominent need for actions aimed at risk reduction. One of the options, although controversial, was the revision of the regulatory standards to be more restrictive and presumably more protective to consumers.

In fact, this option raised the question of whether or not restrictive standards provide adequate protection. To address this question, Wu, Stacy and Kensler [204] calculated the risk of AFB1 in maize and peanut by the deterministic approach using either the same or different carcinogenic potencies of HBsAg^+^ and HBsAg^−^ individuals as those used by JECFA [203]. The seroprevalence of HBsAg^+^ of each country was used to calculate the risk for the corresponding country instead of the conservative 25% generalized by the JECFA on all developing countries. For the consumption pattern of maize and peanut by adults, the authors used data provided by the Proposed Global Environment Monitoring System (GEMS)/Food Consumption Cluster Diets [205]. On this basis, calculations were made for maize and peanut in 91 countries from around the world to determine the maximum allowable levels (MALs) of AFB1 contamination, defined as the contamination levels that would not cause an increase in lifetime HCC risk by more than either 1/100,000 or 1/10,000 individuals in a population [204]. Table 7 shows that, the MTLs in the current regulatory standards in all SEA and African countries are lower than the MALs for the lifetime HCC risk of 1/100,000, and higher, with the exception of Kenya, than the MALs for the lifetime HCC risk of 1/10,000. This suggests that the current standards, when available, in these countries are too relaxed to allow adequate protection if the objective of less than one additional lifetime HCC in 100,000 individuals was sought. Conversely, if the objective of the increase of lifetime HCC were less than one additional case in 10,000 individuals, the risks associated with the consumption of maize and peanut are not of concern to the public health and the current standards provide enough protection. The notable exception of Kenya, which even at the more permissive additional risk of 1/10,000 still had to take further protective measures, emphasized the precariousness of the country’s risk management tools.

Nevertheless, both of the above studies [201,204] concur to suggest that the adoption of highly restrictive standards may not be the ideal solution to provide adequate health protection for consumers. For example, shifting the MTL from 10 to 5 ng/g would allow to increase the daily intake of foods that meet the lower standard only by additional 50 g [201]. Meanwhile, meeting this standard would hamper the productivity of farmers under the present conditions and deter food processors to transform agricultural products in the food industry. Moreover, in such countries where the diet is not varied enough, consumption of the same food repeatedly may exert adverse health effects even if the contamination levels are below the MTL. Similar conclusions were drawn by Wu, Stacy and Kensler [204] whose study demonstrated that SSA countries would have to shift the standards from 20 to <1 ng/g if they consider the option of one additional lifetime HCC risk in 100,000 individuals (Table 9). For the same objective, SEA countries also have to drastically restrict the standards with the consequent management difficulties and uncertainty to provide economically and socially feasible solutions while ensuring adequate protection.

It is worthwhile mentioning that all the risk assessment studies conducted so far on aflatoxins were based on the assumption that they are genotoxic carcinogenic hazards without threshold response and, hence, no NOAEL values to be determined. This assumption has been debated since some authors suggested that aflatoxins have a threshold response [206] which, if confirmed, will change drastically the current perception of aflatoxin risks and urge the revision of previous assessments on a new basis using specifically adapted mathematical models and approaches. In addition, due to the high uncertainties and inaccuracies in dietary intake estimations, there is a tendency to use biomarkers for exposure determinations; yet, the ideal biomarker is still lacking for this purpose, as was discussed earlier [1].

Presently, the biomarker aflatoxin-Lys adduct appears to be the best candidate to estimate chronic exposure. However, adjustments should be made regarding the analytical method to use, the fraction of ingested aflatoxin that binds to albumin, the relationship between the bound fraction and the chronic daily intake for a lifetime, etc. Each of these parameters introduce some uncertainty that affects the accuracy and reliability of the final outcome. Studies on humans in The Gambia and The Peoples’ Republic of China were used to establish a meaningful relationship between the concentration of AFB1-Lys in the serum measured by ELISA technique and the chronic daily intake of aflatoxin for a lifetime period of 70 years. Assuming that an average of 2% of ingested AFB1 binds to albumin, the mean value of 100 pg AFB1–Lys per mg albumin was estimated to correspond to 1 mg/kg bw per day of chronic exposure [207,208]. This biomarker was successfully used to establish the relationship between aflatoxin intake and stunting in children in Benin and Togo, with the weaning diet being a critical factor [209,210,211]. Interestingly, the latter studies demonstrated that regarding the specific toxicological effect of stunting, aflatoxins have a threshold level and 170 ng per kg bw per day did not display a measurable stunting effect [201,211].

It should be pointed out, however, that the exposure evolves (increases or decreases) as function of many key parameters, including diet, agricultural practices, public awareness, climate change, etc. implying that the risk assessment should be revised periodically by national or international expert panels. This is particularly relevant when actions had been taken to manage the risk and the new situation is to be verified for putative improvements. The assessment outcomes discussed herein were considered as the starting point and it is probably worthwhile to conduct new evaluations by national or international organizations with the required expertise. In fact, since the early 2000s, the United Nations through its FAO body has been providing technical and financial assistance to developing countries to reorganize their administrative structures with the risk analysis approach for food safety as the central element. Although many of these countries joined the initiative and made profound administrative changes, the creation of risk assessment bodies or stirring scientific committees within the new administrative organization charts remains challenging. Therefore, JECFA should probably re-assess the risk associated with aflatoxins taking into account the new exposure data and the most recent techniques and models to appraise the present situation and provide recommendations for future actions.

## 6. Current Control Means of Crop Contamination with Aflatoxins

As stated above, the risk associated with the consumption of agri-foods of SEA and SSA countries requires to take actions for safety in order to reduce liver cancer incidence. The action can be done through increased control of crop contamination and/or diet change to reduce aflatoxin intake. Different strategies have been used to reduce the levels and incidence of aflatoxins in foods and agricultural products. These strategies can be split into two main actions: (i) Decontamination by degradation/removal of aflatoxins from crops and (ii) Prevention by the implementation of appropriate management systems to reduce crop contamination in the field and during storage.

### 6.1. Decontamination

Various physical, chemical, and microbial treatments have been demonstrated to reduce crop contamination with aflatoxins by degrading, inactivating or removing them from the matrix [50]. However, each of these treatments has drawbacks that limit its wider application, as it may be onerous, require extreme pressure and temperature conditions, yield inconsistent results, release toxic residues, or reduce the sensory, nutritional or functional properties of the crop [212]. Among the chemical treatments, ozonation and ammoniation have been reported to have received high research attention owing to their effective degradation of aflatoxins and potential for use at large scale. Ozonation of various crops reduced to >99% of the initial level of contamination with aflatoxins in a dose- and time-dependent manner without significantly affecting the chemical composition or the safety of the treated product [213,214]. Technically, it is achieved by blowing gaseous ozone at a given concentration through a layer of the crop to be treated for a given period of time. As a highly oxidative agent, ozone reacts with the furan ring of aflatoxin to breakdown the C_8_ = C_9_ double bond with a consequent inactivation of the mycotoxin, which limits its action to aflatoxins bearing this double bond e.g., AFB1 and AFG1 [215]. In addition to aflatoxin detoxification, ozonation was demonstrated to reduce significantly the counts of moulds including the main aflatoxigenic species *A. flavus* and *A. parasiticus*, thereby preventing the growth of these molds and aflatoxin production during storage [216]. Ammoniation was especially recommended for feed decontamination and could also remove efficiently (>99%) aflatoxins in a dose- and time-dependent manner [217]. As this agent targets the lactone function of the coumarin ring rather than the double bond of the furan ring, it is expected to inactivate a wider range of aflatoxins with similar efficiencies. However, the efficacy this technique increases under high pressure and high temperature and when the moisture of the crop to be treated is within a narrow range (more than 13% and below 16%), which usually requires humidification before treatment followed by drying, thereby adding to the cost of the treatment; and was also reported to decrease the nutritional value of the product [218]. Other innovative technologies claimed to be efficient, cost-effective and environment-friendly have been investigated and continue to attract research interest for wider use [212,219,220].

Techniques to remove aflatoxins from crops by using natural adsorbents that bind to aflatoxins with high affinity, such as clays and organic adsorbents, including microbial strains or their extracts, have been described. However, despite the well-recognized potential of the latter decontamination means, their actual use remains limited essentially due to inconsistent performances; for further details on decontamination technologies, see [218,221]. Difficulties to detoxify aflatoxins is largely related to their chemical nature as a recalcitrant xebobiotic polyheterocyclic toxins that resist biodegradation [222]. Nonetheless, under certain conditions, they can be readily degraded and/or detoxified, usually by disrupting the structure of the terminal furan ring or the coumarin group. Although not toxic by itself, the integrity of the coumarin, in particular its lactone moiety, is necessary for aflatoxins to exert maximum toxicity. Therefore, disruption or removal of the lactone has been associated with drastic reduction or loss of toxicity [223]. This ring is readily opened under extreme pH values (<3 or >10), but can either retrieve its original structure once the pH returns to milder values or be permanently modified if the ring opening is followed by a decarboxylation reaction [224]. The lactone can also be disrupted by different enzymes of various origins, including laccases, peroxidases, reductases, and oxidases [223]. The terminal furan group is another key target for the detoxification of aflatoxins, especially that it is directly involved in the toxicity of many aflatoxins [225]. In recent years, there has been a great interest in aflatoxin-modifying enzymes, which specifically target and modify the terminal furan [226] or the lactone [227] moiety, as a convenient, cost-effective and low risk method to detoxify aflatoxins or generate derivatives with significantly lower toxicities [226,227,228]. In this regard, F_240_-dependent reductases appear to have a great potential as aflatoxin-detoxifying enzymes owing to their high reducing activity on the lactone ring of the toxin and their widespread distribution among members of the *Actinomycetales* order [227]. It also provides feasible and practical alternative to the use of alive microorganisms with inconsistent and unpredictable performances.

### 6.2. Prevention

In view of the above-mentioned limitations of conventional and innovative decontamination methods, there is a general agreement that for these strategies to ensure sustainable protection against aflatoxin contamination, they must be a part of an integrated approach using appropriate quality assurance systems. Such an approach should encompass pre- and post-harvest measures, selection of mold-resistant cultivars, proper use of fungicides and insecticides, adoption of specific cultural techniques depending on the AEZ and increased awareness of the farmers’ regarding the impact of mycotoxin contamination on the crop yield and health [1,86,220]. Among the various actions of this wholistic approach to improve the situation in endemic zones, there is a clear trend to alter the soil microbiome by artificial establishment of non-aflatoxigenic molds strains. In this regard, there is increased research interest in the identification and characterisation of atoxigenic strains of *A. flavus* belonging to vegetative compatibility groups (VCG) that can compete with aflatoxigenic strains and colonize fields where susceptible crops to aflatoxin contamination are cultivated. Such a trend emphasises the need to design appropriate and easy screening and characterization techniques to separate toxigenic from atoxigenic *Aspergillus* strains on the basis of vegetative compatibility analysis. This will help understand the fitness of atoxigenic vs. toxigenic mold strains and their adaptation mechanisms to various environmental and soil conditions in order to adopt effective and environment-friendly biocontrol means, i.e., colonization of fields in various AEZ and soil types by selected atoxigenic strains of different VCGs to displace the naturally occurring toxigenic strains. The first application of this technology was done by the US Agricultural Research Service of the Department of Agriculture (USDA-ARS) in 2003 on cotton using atoxigenic *A. flavus* AF36 strain, which was then registered with the US Environmental Protection Agency (USEPA) [229]. The following year, the same entity patented this technology as a biocontrol product that was licenced by a company under the trade name of afla-guard^®^ [230]. As this technology proved to be an efficient biocontrol means to mitigate aflatoxin contamination in various crops, studies have been conducted in different countries and regions of the world to screen for proficient strains and well adapted to specific soils and AEZs. In Ghana, atoxigenic African *A. flavus* VCG (AAV) strains isolated from three different AEZ reduced aflatoxin contamination of maize and peanut by 87–98% in laboratory assays, and successfully displaced toxigenic *A. flavus* strains in field trials where crops obtained from treated grains contained 50–100% less aflatoxin at harvest than their untreated counterparts [231]. In Northern Italy, co-inoculation of maize ears with an endemic atoxigenic strain *A. flavus* A2085 of the VCG IT019 group and an aflatoxigenic strain (A2092) of the same species was reported to reduce the concentration of AFB1 by 93–98% compared with ears inoculated with the aflatoxigenic strain alone [232]. In field trials, the atoxigenic strain A2085 reduced the concentration of AFB1 in crops at harvest from treated fields by an average of 92.3% compared with the crops from non-treated fields. This strain is now marketed as a biopesticide under the trade name of AF-X1™. Other successful field trials of different scales have been reported in different countries emphasising the anticipated success of this promising technology in the protection of crops against aflatoxin contamination for field to consumption [230,231,233,234].

## 7. Conclusions

This review emphasizes the continuous global health and economic burden of aflatoxins, with SEA and SSA countries supporting the highest share of the burden. Therefore, particular attention should be paid to improvement of the situation in these regions where crops prone to aflatoxin contamination such as peanut, maize, sorghum, and sunflower are grown in agroclimatic zones (hot and humid) favourable to aflatoxin production. Continued high contamination of produce originating from endemic regions is a major hurdle to international trade and to food security. Indeed, this does not affect only local populations, but may extend to other parts of the world by either exporting highly contaminated goods or restricting their marketability, which in turn contribute to increase their prices and limit accessibility to poor social strata. Unfortunately, despite the efforts made in these regions to reduce foods and feeds contamination with aflatoxins, the most recent data suggest that there is no such trend. The incidence and contamination levels vary from one year to another depending mainly on the meteorological conditions, with highest contaminations and incidence recorded in rainy seasons generally proceeded by dry seasons. Yet, the use of atoxigenic strains of *Aspergillus flavus* in the newly developed biocontrol technology to colonize endemic AEZ and displace the aflatoxigenic strains appears to be a promising intervention that should be encouraged and further investigated.

Meanwhile, it appears opportune that the JECFA updates the risk assessment studies to document any positive changes and/or provide informed insights and recommendations for future actions to be taken by a given country or by the international community.

## Figures and Tables

**Table 1:** Origins of aflatoxins and the products most exposed to contamination.

Aflatoxin	Source	Frequently Contaminated Products	References
**Difurocoumarocyclopentenone**			
Aflatoxin B1	Section *Flavi*: *A. flavus, A. pseudotamarii, A. togoensis. A. aflatoxiformans, A. austwickii, A. cerealis, A. arachidicola, A. minisclerotigenes, A. mottae, A. luteovirescens (formerly A. bombycis), A. nomius, A. novoparasiticus, A. parasiticus, A. pipericola, A. pseudocaelatus, A. pseudonomius, and A. sergii, and A. transmontanensis*	Cereals (e.g., sorghum, rice, corn, wheat, barely), oil seeds (e.g., cotton seed, oilseed rape, sunflower seed), nuts (e.g., peanuts, groundnut, pistachio), spices (e.g., turmeric, black and red pepper, ginger, allspices), meats, dairy products, fruit juices, dried fruits, eggs, and feeds and foods derived from these products	[11,14,30,31,32]
	Section *Ochraceorosei*: *A. ochraceoroseus and A. rambellii*		
	Section *Nidulantes: A. astellatus, A. miraensis, A. olivicola, and A. venezuelensis*		
Aflatoxin B2	Section *Flavi*: *A. flavus, A. pseudotamarii, A. aflatoxiformans, A. austwickii, A. cerealis, A. arachidicola, A. minisclerotigenes, A. mottae, A. luteovirescens, A. nomius, A. novoparasiticus, A. parasiticus, A. pipericola, A. pseudocaelatus, A. pseudonomius, A. sergii, A. transmontanensis,* Section *Ochraceorosei: A. ochraceoroseus and A. rambellii*	Cereals (e.g., sorghum, rice, corn, wheat, barely), oil seeds (e.g., cotton seed, oilseed rape, sunflower seed), nuts (e.g., peanuts, groundnut, pistachio), spices (e.g., turmeric, black and red pepper, ginger), meats, dairy products, fruit juices, dried fruits, eggs, and feeds and foods derived from these products	[11,14,30,31,32]
Aflatoxin B2_a_	Hydroxylated metabolite of aflatoxin B1 obtained by water addition to the double bond of the terminal furan under acidic conditions in the liver, the stomach or soil (no evidence for the involvement of specific enzymes)	NA	[33,34,35,36]
	Naturally produced by *A. flavus*, and *A. parasiticus*		
Aflatoxin M1	Hydroxylated metabolite of aflatoxin B1 by hepatic microsomal mixed-function oxidase system (MFO), mainly cytochromes, in the liver of mammals Produced in vitro from aflatoxin B1 by liver homogenates Naturally produced by *A. flavus* and *A. parasiticus*	Milk (including human milk) and dairy products Meat products (kidney, liver) Mouldy groundnut and corn	[30,37,38]
Aflatoxin M2	Hydroxylated metabolite of B2 by hepatic microsomal MFO of mammals Naturally produced by *A. parasiticus*	Idem as aflatoxin M1	[30,38]
Aflatoxin M2_a_	Hydration of the terminal furan ring of aflatoxin M1 in dilute acid to yield an hemiketal derivative In vitro in liver homogenates	Milk and dairy products	[39]
Aflatoxin P1	Demethylated metabolite of aflatoxin B1 by liver microsomal oxidase–catalysed O-demethylase	Mainly excreted in the urine (humans and animals). Dairy products	[30,37,40,41]
Aflatoxin Q1	Hydroxylated metabolite of aflatoxin B1 by microsomal enzymes in the liver of higher vertebrates and poultry (main aflatoxin B1 metabolite in monkey)	Assumed to be in edible parts of bovine fed on aflatoxin B1-contaminated feed	[30,37,42]
Aflatoxin Q2_a_	Acid hydration of aflatoxin Q1	NA	[43]
Aflatoxicol (R_0_)	Metabolite of aflatoxin B1 formed by a reversible reduction of the pentanone group in humans, animals and numerous bacteria and molds In vitro biotransformation of aflatoxin B1 by a soluble cytoplasm reductase system in fish, rat and human liver preparations Naturally produced by *A. flavus* and *A. parasiticus*	Mainly avian products (major metabolite in avian species fed on B1-contaminated feed). Dairy products Does not accumulate in edible parts of bovine and swine fed on aflatoxin B1-contaminated feed	[40,41,44,45,46,47,48,49,50]
Aflatoxicol M1	Reduced metabolite of aflatoxin B1, aflatoxin R_0_, or aflatoxin M1 catalysed by soluble NADPH-dependent reductases in the liver	Milk and dairy products	[30]
Aflatoxicol H1	Reduced metabolite of aflatoxin B1 and aflatoxin Q1 catalysed by soluble NADPH-dependent reductases in the liver	Milk and dairy products	[30,51]
**Difurocoumarolactone**			
Aflatoxin G1	*A. flavus* ^a^ *, A. aflatoxiformans, A. austwickii, A. cerealis, A. arachidicola, A. minisclerotigenes, A. mottae, A. luteovirescens, A. nomius, A. novoparasiticus, A. parasiticus, A. pipericola, A. pseudocaelatus, A. pseudonomius, A. sergii, A. transmontanensis,*	Cereals (e.g., sorghum, rice, corn, wheat, barely), oil seeds (e.g., cotton seed, oilseed rape, sunflower seed), nuts (e.g., peanuts, groundnut, pistachio), spices (e.g., turmeric, black and red pepper, ginger), meats, dairy products, fruit juices, dried fruits, eggs, and feeds and foods derived from these products	[11,14,30,31,32]
Aflatoxin G2	*A. flavus^1^, A. aflatoxiformans, A. austwickii, A. cerealis, A. arachidicola, A. minisclerotigenes, A. mottae, A. luteovirescens, A. nomius, A. novoparasiticus, A. parasiticus, A. pipericola, A. pseudocaelatus, A. pseudonomius, A. sergii, and A. transmontanensis*	Same as aflatoxin G1	[11,14,30,31,32]
Aflatoxin G2_a_	Hydroxylated metabolite of aflatoxin G1 obtained by catalytic addition of water to the double bond of the terminal furan under acidic conditions in the liver, the stomach or soil (no evidence for the involvement of specific enzymes). Naturally produced by *A. flavus*	NA	[30,35,36]
Aflatoxin GM1	Hydroxylated metabolite of aflatoxin G1 by MFO in the liver of mammals Produced in vitro by *A. parasiticus* fed aspertoxin as a precursor Naturally produced by *A. flavus*	Milk and dairy products	[38,39,52]
Aflatoxin GM2	Hydroxylated derivative of aflatoxin G2 by MFO in the liver of mammals Produced in vitro by *A. parasiticus* from dihydro-O-methylsterigmatocystin (DHOMST) Naturally produced by *A. flavus* and *A. parasiticus* and yeast	Milk and dairy products	[38,39]
Aflatoxin GM2_a_	Metabolite of aflatoxin GM1in the liver of mammals Hydration of the terminal furan ring of aflatoxin M1 in dilute acid to yield an hemiketal in vitro in liver homogenates	Milk and dairy products	[39]
Parasiticol (aflatoxin B3)	*A metabolite of aflatoxin G1 from the biodegradation (hydrolysis and decarboxylation reactions) in A. flavus, Rhizopus stolonifer, Rhizopus arrhizus, and Rhizopus oryzae*	Idem as aflatoxins B1 and G1	[14,52,53,54,55]
**Others**			
Parasiticol (aflatoxin B3)	A metabolite of aflatoxin G1 from the biodegradation (hydrolysis and decarboxylation reactions) in *A. flavus, Rhizopus stolonifer, Rhizopus arrhizus*, and *Rhizopus oryzae* Naturally produced by *A. parasiticus, A. flavus, A. mottae, A. nomius, and A. novoparasiticus*	Idem as aflatoxins B1 and G1	[14,52,53,54,55]
Aspertoxin ^b^	*A. flavus and A. parasiticus*	Mainly vegetal products prone to contamination with *A. flavus* and *A. parasiticus*; not considered to be relevant to food products of animal origin	[38,56]

^a^ Not a typical producer of G-types of aflatoxins, but some strains were reported to produce them in addition to B1 and B2 [14]; ^b^ Usually considered as a sperate mycotoxin produced by *A. flavus* because of structural differences with the difurocoumarin structure that characterizes the aflatoxins. Abbreviations: NA: Not available.

**Table 2:** Key properties of aflatoxins and their metabolites. Data compiled from PubChem of the National Center for Biotechnology Information [57]) and ChemSpider of the Royal Society of Chemistry [58] databases, unless references are indicated beside the data.

Aflatoxin	MW (g/mol)	Formula	Melting Point (°C) ^a^	Toxicity			Adverse Health Effects ^b^
				LD_50_ (mg/kg bw)	Test Organism	Route	
Aflatoxin B1	312.063	C_17_H_12_O_6_	268.5	0.24–60 [59] 3.0	Various animals and chick embryo Human	Oral, intraperitoneal or injection in chick embryo In vitro experiments	Hepatotoxicity, genotoxicity, carcinogenicity, immuno-toxicity, teratogenicity
Aflatoxin B2	314.079	C_17_H_14_O_6_	286–289 [59]	1.7	Duck	Oral	Week mutagenicity, hepatotoxicity, and carcinogenicity [45]
Aflatoxin B2_a_	330.074	C_17_H_14_O_7_	240 [59]	>400 μg showed a weak toxicity [60,61]	Ducklings	Oral	Low toxicity (200-fold less than B1) [34,61]
Aflatoxin M1	328.058	C_17_H_12_O_7_	297–299	0.32 1.5	Duck Rat	Unreported Oral	Hepatotoxicity, nephrotoxicity, carcinogenicity
Aflatoxin G2	330.074	C_17_H_14_O_7_	237–240 226–229	2.5 [39] Weekly mutagenic	Duckling *S. typhimurium*	Oral Ames’ test	Low toxicity, no evidence for carcinogenicity in animals [30,45,62]
Aflatoxin G2_a_^c^	346.069	C_17_H_14_O_8_	243.13 (Predicted)	NA	NA	NA	Low toxicity to inactive (a detoxified form of G1) [30,45]

^a^ Data collected from ChemSpider website (http://www.chemspider.com) unless indicated by an imbedded citation; ^b^ In the latest classification of mycotoxins, the IARC stated that there is “sufficient evidence” for the carcinogenicity of aflatoxins B1, G1, and M1 in experimental animals, but there is “limited evidence” or “insufficient evidence” in experimental animals for the carcinogenicity of aflatoxins B2 and G2, respectively; however, in view of mechanistic studies showing the ability of the major aflatoxins (B1, G1, B2, G2, M1) to form DNA adducts as a first step in genotoxicity, they were classified in group 1 carcinogens [62]; ^c^ Mutagenicity induced in *Salmonella typhimurium* is <1% that of aflatoxin B1 taken as a reference [45]. *Abbreviations:* NA: Not available.

**Table 3:** Climatic conditions in countries reputed for their vulnerability to aflatoxin contamination. Data are mean values for the period of 1901–2016 [101,102].

Region Country	Annual Temperature (°C)			Mean Annual Rainfall (mm)	Predominating Climate Types ^a^
	Min	Max	Mean		
Sub-Saharan Africa					
Benin	25.3	30.3	27.5	1059	Tropical savanna (Aw)
Cameroun	23.4	26.7	24.8	1614	Tropical savanna (Aw)
Ghana	25.3	29.5	27.3	1190	Tropical savanna (Aw)
Kenya	22.6	25.9	24.3	669	Tropical savanna (Aw)
Mali	21.2	33.4	28.3	333	Tropical savanna (Aw)
Nigeria	18.5	32.4	25.4	881	Tropical savanna (Aw)
Tanzania	19.9	23.5	22.2	998	Tropical savanna (Aw)
Togo	25.0	29.5	27.0	1170	Tropical savanna (Aw)
Uganda	21.3	23.6	22.4	1200	Tropical savanna (Aw)
Zambia	17.2	25.0	22.0	976	Humid subtropical (Cwa)
South Africa	14.6	25.9	20.3	779	Temperate oceanic (Cfb)
Southeast Asia					
India	17.0	30.0	24.1	1057	Tropical savanna (Aw)
Indonesia	22.8	30.2	28.9	2859	Tropical rainforest (Af)
Malaysia	24.9	25.9	25.4	3059	Tropical rainforest (Af)
Philippines	24.3	27.0	25.5	2471	Tropical rainforest (Af)
Thailand	23.0	28.9	26.3	1553	Tropical savanna (Aw)
Vietnam	20.0	27.22	26.0	149.4	(Aw)

^a^ In the same country, there are generally more than one climate type depending on the geographical region, which is defined as agroecological zone (AEZ) according the classification of Köppen-Geiger [103], where the first letter refers to the climate type (A: Tropical; B: Arid; C: Warm temperate), the second letter refers to the precipitation (w: Winter dry; S: Steppe; f: Fully humid; m: Monsoonal), and the third letter refers to the temperature (h: hot arid; a: Hot summer; b: Warm summer).

**Table 4:** Minimum (Min), maximum (Max) and optimum (Opt) values of Temperature (°C) and water activity (a_w_) for the growth and aflatoxin production by *Aspergillus flavus* and *Aspergillus parasiticus* in selected grains and in laboratory media.

Substrate/ Parameter	Growth						Aflatoxin Production						References
	*A. flavus*			*A. parasiticus*			*A. flavus*			*A. parasiticus*			
	Max	Min	Opt	Max	Min	Opt	Max	Min	Opt	Max	Min	Opt	
Wheat/													
Temperature	>42.5	15	35	-	-	-	42.5	15.0	25	-	-	-	[104]
a_w_	>0.95	0.80	0.95	-	-	-	0.95	0.85	0.93	-	-	-	
Nyjer seeds ^a^/													
Temperature	NS	NS	27	NS	NS	27	NS	20	27.0	NS	20	27.0	[105]
a_w_	NS	0.82	0.98	NS	0.82	0.94	NS	0.86	0.90	NS	0.86	0.98	
Sorghum/													
Temperature	NS	15	37	-	-	-	NS	15	37	-	-	-	[106]
a_w_	NS	<0.91	0.97	-	-	-	NS	0.94	0.97	-	-	-	
Rice/													
Temperature	42	20	33	-	-	-	37	<20	35	-	-	-	[107]
a_w_	0.99	0.80	0.90	-	-	-	0.99	0.85	0.96	-	-	-	
Sabouraud/													
Temperature	NS	0.90	0.99	-	-	-	NS	0.90	0.99	-	-	-	[108]
a_w_	NS	15	NS	-	-	-	NS	15	NS	-	-	-	
Malt Extract-Sucrose/													
Temperature	-	-	-	42	15	35	-	-	-	40 ^b^ 37 ^c^	17 ^b^ 17 ^c^	37 ^b^ 20 ^c^	[109]
	NS	12	37	NS	13	32	37	12	31	NS	10–13	24	[110,111]
	42	15	30–35	-	-	-	-	-	-	35	<20	25	[70]
a_w_	-	-	-	NS	0.90	0.99	-	-	-	NS NS	0.90 ^b^ 0.90 ^c^	0.93 ^b^ 0.99 ^c^	[109]
	NS	0.80	0.99	NS	0.83	>0.99	NS	0.85	0.99	NS	0.91	0.99	[110,111]
	NS	0.85	0.99	-	-	-	0.99	0.85	0.99	-	-	-	[70]

^a^ Scientific name *Guizotia abyssinica*, also called thistle or Niger seeds extensively used in Sub-Sharan region to extract oil, ^b^ For aflatoxin B1 production, ^c^ For aflatoxin G1 production. Abbreviation: NS: not specified

**Table 5:** Incidence (%) and concentrations (μg/kg) of aflatoxins in staple agricultural products of selected countries from Sub-Saharan Africa. Data are for total aflatoxins (B1 + B2 + G1 + G2), unless otherwise stated in the footnotes of Supplementary Material Tables S2 and S3.

Country	Climate Type ^a^	Peanut/Groundnut		Maize		Millet		Sorghum		Sunflower		References
		Mean Range ^b^ (Min–Max)	+ve (%)	Mean Range ^b^ (Min–Max)	+ve (%)	Mean Range ^b^ (Min–Max)	+ve (%)	Mean Range ^b^ (Min–Max)	+ve (%)	Mean Range ^b^ (Min–Max)	+ve (%)	
Uganda	Am, Aw, Af	7.3–221 (2.5–849)	20–82	25.4–75.2 (3.1–510)	50–100	14.0 (NS–NS)	100	11.5–170 (4–472)	80–100	-	-	[79,124,125]
Kenya	Cbw, Afa, Cfb, Am, Cfb, Aw, As, Cwb, BSh	<LD-1140 (0.1–32,328) ^c^	7.5–100	0.7, 196.3 (0–48,000)	25–98	0.1–66 (1.0–1658)	21–98	0.9–24.5 (<1–265)	11–100	1524 (NS–NS)	NS	[21,84,86,99,122,126,127,128,129,130,131,132,133]
Tanzania	Cwb, Aw, Csb, Bsh, Cfa	0–377.3 (56–3297)	NS	0.76–106 (1–1081)	4–28	-	-	2.7–93.3 (0–138.7)	NS	4.9–119 1.4–663	50–89	[100,134,135,136,137]
Zambia	Aw, Cwa, BSh,	4.43–499 (3.9–11,100)	51–100	11–12.0 (3.9–3420)	42–73	-	-	-	-	-	-	[7,24]
South Africa	Cwb, BSh, Cfb	6.0–14(74–1416)	27 ^d^–90	1–48 (1–1416)	6.5–22 ^d^	-	-	-	-	-	-	[113,138,139]
Nigeria	BSh, Aw, Am	6.0–96 (0.9–646)	26	0.6–603 (2.7–1460)	10–87.5	34.3–120.5 (NS–NS)	NS	165–1245 (0–1164)	45--64			[80,82,121,140,141,142]
Cameroun	Am, Af, Aw	22–26 (6.0–125)	NS	47–100 (6–645)	NS	-	-	-	-	-	-	[143]
Ghana	Aw, HF, DS SGS, FRT, SVT, RFR	0.3–145.6 (17–3868)	NS	6–341 (1–945)	NS 42,100	- -	- -	14 (6–19)	- 25	- -	- -	[144,145,146]
Togo	Aw, DS, SGS	0.3–34.9 (0–168)	NS	6.8–24.2 (0–157)	-	-	-	-	-	-	-	[145]
Benin	Aw	7.6 (<0.1–105)	19	1.6 (<0.1–20)	32	-	-	-	-	-	-	[80]
Mali	Aw	2.2–9.4 (<0.1–246)	15–29	-	-	-	-	-	-	-	-	[80]
Mozambique	Aw	5.9–751 (0–2740)	8.3–92.0	2.4–22 (NS-NS)	67	-	-	-	-	-	-	[147,148,149]

^a^ The type of climate in the agroecological zones (AEZs) where crops are grown as defined by Köppen-Geiger classification (http://koeppen-geiger.vu-wien.ac.at): Cfb: Warm temperate (C) fully humid (f) warm summer (b); Cwa: Warm temperate (C) winter dry (w) hot summer (a); Cwb: Warm temperate (C) winter dry (w) warm summer (b); Af: Tropical (A) fully humid (f); Aw: Tropical (A) winter dry (w); As: Tropical (A) steppe (s); Am: Tropical (A) monsoonal (m), Csb: Warm temperate (C) steppe (s) warm summer (b); BSh: Arid (B) steppe (S) hot (h), Cfa: Warm temperate (C) fully humid (f) hot summer (a), ^b^ Arithmetic mean or mean range as default or geometric mean as specified in the Supplementary Materials Tables S2 and S3; ^c^ Exceptionally high aflatoxin levels recorded in 2004 during a major aflatoxicosis in Kenya; ^d^ Percentages were calculated for samples containing more than 4.0 µg/kg of aflatoxins. *Abbreviations and symbols*: AEZ: Argo-ecological zone; Min: Minimum; Max: Maximum; +ve: Positive samples (aflatoxin levels higher than the level of detection LOD unless specified otherwise); “-“: no available data, 0 “zero”: aflatoxin level below LOD; NS: Not specified; HF = Humid Forest, DS = Derived Savanna, and SGS = Southern Guinea Savanna. SVT = Savana Transition; RFR = Rain Forest; FRT = Forest Transition.

**Table 6:** Incidence and concentrations (μg/kg) of aflatoxin contamination of staple crops in selected countries from the Southeast Asian region. Data are for total aflatoxins (B1 + B2 + G1 + G2), unless otherwise stated in the footnotes.

Country	AEZ (Climate Type) ^a^	Peanut/Groundnut		Maize		Rice		Sorghum		References
		Mean Range ^b^ (Min–Max)	+ve (%)	Mean Range (Min–Max)	+ve (%)	Mean Range (Min-Max)	+ve (%)	Mean Range (Min-Max)	+ve (%)	
India	BSh, Aw, Cwa, Am, BWh, Af	510.7 (NS–NS)	NS	5–67.3 (0–714)	21–100	NS (0.1–308)^c^	68–91	882 ^c^ (0.01–1250)	82–100	[150,151,152,153,154]
Nepal	Eastern region (Cfa)	NS (54–1806)	34	NS (64–859)	32	-	-	-	-	[155]
The Philippines	Af, Aw	58 (0–885)	65	39–76 (0.0–1215)	95	1.5 (0–8.7)	95	-	-	[156,157,158]
Thailand	Aw, Am, Af	28–1811 (0–12,256)	43–91	196–400 (0–2730)	39	0.8–67 (0–248)	2–63	-	-	[72,119,120,159,160,161]
Malaysia	NS	4.3 ^c^–11.3 (1.5–1000)	16–85	-	-	1.75 ^c^ (1.1–5.2)	25–70	-	-	[162,163,164,165,166]
Indonesia	Aw, Af	-	-	144–464 (NS–490)	92–100	-	-	-	-	[163]
Vietnam	Aw	4.96–16.57 (>0.1–362)	21–26	2.62–66.1 (>0.1–1572)	30–35	0.42–2.04 (>0.1–93)	5.4–12.5	-	-	[81]

Captions and abbreviations are as defined in the footnotes of Table 5.

**Table 7:** Significant aflatoxicosis outbreaks in Asia and Africa.

Country	Region	Year (Period)	Number of Cases	Number of Deaths (% of Fatality Rate)	Associated Food	Level of Contamination (mg/kg)	Specific Remark	Reference
India	Western (Rajasthan-Gujarat)	1974 (October–November)	397	106 (26.7)	Maize	6.3–15.6	Heavy unseasonal rain after drought and faulty storage conditions	[116]
Kenya	East-Central(Makueni)	1981 (March–June)	20	12 (60)	Maize	3.2 and 12.0	Rain shortage in the year preceding the outbreak followed by prolonged high rainy season and faulty storage conditions	[85]
	East-Central (Makueni-Kitui-Machakos-Thika)	2004 (January–July) ^a^	317	125 (39.4)	Maize	1.0–46.4	First study relating aflatoxin-albumin adduct to human aflatoxicosis and its use as a biomarker	[84,193]
Malaysia	Perak state	1988	17 ^b^	13 ^c^ (76.5)	Chinese noodles ^d^	NS	Possible additive effect of boric acid and aflatoxin	[182,185]
Tanzania	Central	2016 (May–November)	67	20 (30)	Maize	10–51,100	Possible additive effect of fumonisins High titres of aflatoxin-albumin adduct in the serum of patients used as evidence for the aflatoxicosis	[183]
	Northeast	2017 (June–July)	8	4 (50)	Maize	NA	Evidence based on symptoms and consumption of maize reported to have been inadequately stored	[194]

^a^ A peak was reached in May to Mid-July; ^b^ 16 children 2.5–11 years of age a 49-years old adult; ^c^ All children, and they died within hours of the intoxication and the percentage of death excludes 45 cases not admitted to the hospital because they developed only mild symptoms; ^d^ Dish called “Loh See Fun” suspected to have been preserved with banned boric acid. Abbreviations: BAP: Boric acid poisoning; the others as in the Tables above.

**Table 8:** Risk for primary liver cancer (hepatocellular carcinoma) in some African countries determined by the deterministic approach as function of the exposure from different food products and the carcinogenic potency or by the margin of exposure (MOE) using the benchmark dose (BMD) approach. Adapted from [201].

Country	Product	Exposure ^a^	R ^b^	MOE ^c^
Kenya	Maize (commercial)	133	11	1.3
	Maize (rural market)	353	29.2	0.5
Ghana	Kenkey ^d^	850	70.1	0.2
Botswana	Peanut butter	23	1.9	7.4
Benin	Yam chips ^e^	105	8.7	1.6
The Gambia	Maize	3.6	0.3	47.2
	Millet	30	2.4	5.7
	Sorghum	1.4	0.1	121.4
	Rice	14	1.1	12.1
	Groundnut	16	1.3	10.6

^a^ Calculated on the basis the highest dietary intake recorded in each country; ^b^ Risk for primary liver cancer per 10^5^ individuals per year [200]; ^c^ MOE defined as the ratio between benchmark dose limit of 10% extra risk (BMDL10) and the exposure dose [203]; the BMDL10 is determined experimentally on rodents as 170 ng/kg body weight/day; ^d^ Fermented maize product; ^e^ Chips made from blends of sweet potato and maize.

**Table 9:** The maximum allowable levels (MALs) of AFB1 in maize and peanut as calculated by deterministic approach using the carcinogenic potency of JECFA, with adjusted prevalence of seropositive for HB, and exposure from the cluster diet as provided by the World Health Organization [205]. Adapted from [204].

Cluster Diet	Country	MTL ^a^ (ng/g)	MAL ^b^ (ng/g)	
			Risk 1/100,000	Risk 1/10,000
G	China	40	7	66
	India	30	13	129
	Indonesia	20	13	127
	Malaysia	15 ^c^	9	89
	Nepal	40	17	169
	Sri Lanka	NA	14	138
	Thailand	NA	9	94
	Vietnam	NA	7	66
I	Kenya	20	1	9
	Malawi	10	1	12
	Mozambique	10 ^b^	2	17
	South Africa	10	2	20
	Tanzania	10	2	16
	Zimbabwe	10	1	10
J	Nigeria	NA	2	24
	Sudan	NA	4	42
L	The Phillippines	20	7	68

^a^ Current official standards for MTL in peanut and maize; ^b^ maximum allowable levels calculated as the maximum levels of AFB1 contamination that would cause a lifetime risk increase of one case HCC in 10,000 or 100,000 individuals in a population; ^c^ Standard for peanut only. *Abbreviations:* NA: Not available; HB: Hepatitis virus B; JECFA: The joint Food and Agriculture Organization (FAO)/World Health Organization (WHO) Expert Committee on Food Additives and Contaminants.

## Supplementary Materials

The following are available online at https://www.mdpi.com/1660-4601/17/4/1215/s1, Table S1: Key properties of aflatoxins and their metabolites. Data compiled from PubChem of the National Center for Biotechnology Information (https://pubchem.ncbi.nlm.nih.gov) and ChemSpider of the Royal Society of Chemistry (http://www.chemspider.com) databases, unless references are indicated beside the data, Table S2: Incidence (%) and concentrations (µg/kg) of aflatoxins in staple agricultural products of selected countries from Sub-Saharan Africa. Data are for total aflatoxins (B1+B2+G1+G2), unless otherwise stated in the footnotes, Table S3: Incidence and concentrations (µg/kg) of aflatoxin contamination of staple crops in selected countries from the Southeast Asian region. Data are for total aflatoxins (B1+B2+G1+G2), unless otherwise stated in the footnotes.

## References

[1] Benkerroum N. (2019). Retrospective and prospective look at aflatoxin research and development from a practical standpoint. Int. J. Environ. Res. Public Health.

[2] Benkerroum N. (2016). Mycotoxins in dairy products: A review. Int. Dairy J..

[3] Bhat R., Rai R.V., Karim A.A. (2010). Mycotoxins in food and feed: Present status and future concerns. Compr. Rev. Food Sci. Food Safety.

[4] Hymery N., Vasseur V., Coton M., Mounier J., Jany J.-L., Barbier G., Coton E. (2014). Filamentous fungi and mycotoxins in cheese: A review. Compr. Rev. Food Sci. Food Safety.

[5] Rohlfs M. (2014). Fungal secondary metabolite dynamics in fungus-grazer interactions: novel insights and unanswered questions. Front. Microbiol..

[6] Pusztahelyi T., Holb I.J., Pocsi I. (2015). Secondary metabolites in fungus-plant interactions. Front Plant Sci..

[7] Njoroge S.M.C., Matumba L., Kanenga K., Siambi M., Waliyar F., Maruwo J., Machinjiri N., Monyo E.S. (2017). Aflatoxin B1 levels in groundnut products from local markets in Zambia. Mycotoxin Res..

[8] Nielsen K.F., Smedsgaard J. (2003). Fungal metabolite screening: database of 474 mycotoxins and fungal metabolites for dereplication by standardised liquid chromatography–UV–mass spectrometry methodology. J. Chromatogr. A.

[9] IARC (International Agency for Research on Cancer) (2002). Some traditional herbal medicines, some mycotoxins, naphthalene and styrene. IARC Monographs on the Evaluation of Carcinogenic Risks to Humans.

[10] Liu Y., Wu F. (2010). Global burden of aflatoxin-induced hepatocellular carcinoma: a risk assessment. Environ. Health Perspect..

[11] Pildain M.B., Frisvad J.C., Vaamonde G., Cabral D., Varga J., Samson R.A. (2008). Two novel aflatoxin-producing *Aspergillus* species from Argentinean peanuts. Int. J. Syst. Evol. Microbiol..

[12] Okoth S., De Boevre M., Vidal A., Diana Di Mavungu J., Landschoot S., Kyallo M., Njuguna J., Harvey J., De Saeger S. (2018). Genetic and toxigenic variability within *Aspergillus flavus* population isolated from maize in two diverse environments in Kenya. Front. Microbiol..

[13] Gilbert M.K., Mack B.M., Moore G.G., Downey D.L., Lebar M.D., Joardar V., Losada L., Yu J., Nierman W.C., Bhatnagar D. (2018). Whole genome comparison of *Aspergillus flavus* L-morphotype strain NRRL 3357 (type) and S-morphotype strain AF70. PLoS ONE.

[14] Frisvad J.C., Hubka V., Ezekiel C.N., Hong S.B., Nováková A., Chen A.J., Arzanlou M., Larsen T.O., Sklenář F., Mahakarnchanakul W. (2019). Taxonomy of *Aspergillus* section *Flavi* and their production of aflatoxins, ochratoxins and other mycotoxins. Stud. Mycol..

[15] Abdin M.Z., Ahmad M.M., Javed S. (2010). Advances in molecular detection of *Aspergillus*: An update. Arch. Microbiol..

[16] Bennett J.W., Klich M. (2003). Mycotoxins. Clin. Microbiol. Rev..

[17] Nesbitt B.F., O’Kelly J., Sargeant K., Sheridan A.N.N. (1962). *Aspergillus flavus* and turkey X disease: Toxic metabolites of *Aspergillus flavus*. Nature.

[18] Buchanan Jr R.L., Ayres J.C. (1976). Effect of sodium acetate on growth and aflatoxin production by *Aspergillus parasiticus* NRRL 2999. J. Food Sci..

[19] Wicklow D.T., Shotwell O.L. (1983). Intrafungal distribution of aflatoxins among conidia and sclerotia of Aspergillus flavus and Aspergillus parasiticus. Can. J. Microbiol..

[20] Ehrlich K.C., Chang P.K., Yu J., Cotty P.J. (2004). Aflatoxin biosynthesis cluster gene cypA is required for G aflatoxin formation. Appl. Environ. Microbiol..

[21] Mutegi C.K., Cotty P.J., Bandyopadhyay R. (2018). Prevalence and mitigation of aflatoxins in Kenya (1960-to date). World Mycotoxin J..

[22] Probst C., Callicott K.A., Cotty P.J. (2012). Deadly strains of Kenyan *Aspergillus* are distinct from other aflatoxin producers. Eur. J. Plant Pathol..

[23] Geiser D.M., Dorner J.W., Horn B.W., Taylor J.W. (2000). The phylogenetics of mycotoxin and sclerotium production in *Aspergillus flavus* and *Aspergillus oryzae*. Fungal Genet. Biol..

[24] Kachapulula P.W., Akello J., Bandyopadhyay R., Cotty P.J. (2017). Aflatoxin contamination of groundnut and maize in Zambia: observed and potential concentrations. J. Appl. Microbiol..

[25] Chang P.-K., Bennett J.W., Cotty P.J. (2002). Association of aflatoxin biosynthesis and sclerotial development in *Aspergillus parasiticus*. Mycopathologia.

[26] Probst C., Schulthess F., Cotty P.J. (2010). Impact of *Aspergillus* section *Flavi* community structure on the development of lethal levels of aflatoxins in Kenyan maize (Zea mays). J. Appl. Microbiol..

[27] Townsend C.A. (2014). Aflatoxin and deconstruction of type I, iterative polyketide synthase function. Nat. Prod. Rep..

[28] Yu J., Chang P.-K., Ehrlich K.C., Cary J.W., Bhatnagar D., Cleveland T.E., Payne G.A., Linz J.E., Woloshuk C.P., Bennett J.W. (2004). Clustered pathway genes in aflatoxin biosynthesis. Appl. Environ. Microbiol..

[29] Theumer M.G., Henneb Y., Khoury L., Snini S.P., Tadrist S., Canlet C., Puel O., Oswald I.P., Audebert M. (2018). Genotoxicity of aflatoxins and their precursors in human cells. Toxicol. Lett..

[30] Deshpande S. (2002). Fungal toxins. Handbook of Food Toxicology.

[31] Khazaeli P., Mehrabani M., Heidari M.R., Asadikaram G., Lari Najafi M. (2017). Prevalence of aflatoxin contamination in herbs and spices in different regions of Iran. Iran J. Public Health.

[32] Chen A.J., Frisvad J.C., Sun B.D., Varga J., Kocsubé S., Dijksterhuis J., Kim D.H., Hong S.B., Houbraken J., Samson R.A. (2016). *Aspergillus* section Nidulantes (formerly Emericella): Polyphasic taxonomy, chemistry and biology. Stud. Mycol..

[33] Rushing B.R., Selim M.I. (2016). Effect of dietary acids on the formation of aflatoxin B2a as a means to detoxify aflatoxin B1. Food Addit. Contam. Part A.

[34] Dutton M.F. (1988). Enzymes and aflatoxin biosynthesis. Microbiol. Rev..

[35] Starr J.M., Rushing B.R., Selim M.I. (2017). Solvent-dependent transformation of aflatoxin B1 in soil. Mycotoxin Res..

[36] Swick R.A. (1984). Hepatic metabolism and bioactivation of mycotoxins and plant toxins. J. Anim. Sci..

[37] Hayes J.R., Polan C.E., Campbell T.C. (1977). Bovine liver metabolism and tissue distribution of aflatoxin B1. J. Agric. Food Chem..

[38] Yabe K., Chihaya N., Hatabayashi H., Kito M., Hoshino S., Zeng H., Cai J., Nakajima H. (2012). Production of M-/GM-group aflatoxins catalyzed by the OrdA enzyme in aflatoxin biosynthesis. Fungal Genet. Biol..

[39] Applebaum R.S., Brackett R.E., Wiseman D.W., Marth E.H. (1982). Aflatoxin: Toxicity to dairy cattle and occurrence in milk and milk products—A review. J. Food Prot..

[40] Trucksess M.W., Stoloff L., Brumley W.C., Wilson D.M., Hale O.M., Sangster L.T., Miller D.M. (1982). Aflatoxicol and aflatoxins B1 and M1 in the tissues of pigs receiving aflatoxin. J. Assoc. Off. Anal. Chem..

[41] Carvajal-Moreno M., Vargas-Ortiz M., Hernández-Camarillo E., Ruiz-Velasco S., Rojo-Callejas F. (2019). Presence of unreported carcinogens, aflatoxins and their hydroxylated metabolites, in industrialized Oaxaca cheese from Mexico City. Food Chem. Toxicol..

[42] Rawal S., Coulombe R.A. (2011). Metabolism of aflatoxin B1 in turkey liver microsomes: The relative roles of cytochromes P450 1A5 and 3A37. Toxicol. Appl. Pharmacol..

[43] Fan T.S., Zhang G.S., Chu F.S. (1984). Production and characterization of antibody against aflatoxin Q1. Appl. Environ. Microbiol..

[44] Nakazato M., Morozumi S., Saito K., Fujinuma K., Nishima T., Kasai N. (1990). Interconversion of aflatoxin B1 and aflatoxicol by several fungi. Appl. Environ. Microbiol..

[45] Wong J.J., Hsieh D.P. (1976). Mutagenicity of aflatoxins related to their metabolism and carcinogenic potential. Proc. Natl. Acad. Sci. USA.

[46] Salhab A.S., Edwards G.S. (1977). Production of aflatoxicol from aflatoxin B1 by postmitochondrial liver fractions. J. Toxicol. Environ. Health.

[47] Nakazato M., Saito K., Kikuchi Y., Ibe A., Fujinuma K., Nishijima M., Nishima T., Morozumi S., Wauke T., Hitokoto H. (1985). Aflatoxicol formation by *Aspergillus flavus* and *A. parasiticus*. Food Hyg. Safey Sci..

[48] Detroy R.W., Hesseltine C.W. (1969). Transformation of aflatoxin B1 by steroid-hydroxylating fungi. Can. J. Microbiol..

[49] Frazzoli C., Gherardi P., Saxena N., Belluzzi G., Mantovani A. (2017). The hotspot for (Global) one health in primary food production: Aflatoxin M1 in dairy products. Front Public Health.

[50] Doyle M.P., Applebaum R.S., Brackett R.E., Marth E.H. (1982). Physical, chemical and biological degradation of mycotoxins in foods and agricultural commodities. J. Food Prot..

[51] Salhab A.S., Hsieh D.P. (1975). Aflatoxicol H1: A major metabolite of aflatoxin B1 produced by human and rhesus monkey livers in vitro. Res. Commun. Chem. Pathol. Pharmacol..

[52] Heathcote J.G., Dutton M.F. (1969). New metabolites of Aspergillus flavus. Tetrahedron.

[53] Stubblefield R.D., Shotwell O.L., Shannon G.M., Weisleder D., Rohwedder W.K. (1970). Parasiticol: A new metabolite from *Aspergillus parasiticus*. J. Agric. Food Chem..

[54] Cole R.J., Kirksey J.W. (1971). Aflatoxin G1 metabolism by Rhizopus species. J. Agric. Food Chem..

[55] Ji C., Fan Y., Zhao L. (2016). Review on biological degradation of mycotoxins. Anim. Nutr..

[56] Rodricks J.V., Lustig E., Campbell A.D., Stoloff L. (1968). Aspertoxin, a hydroxy derivative of O-methylsterigmatocystin from aflatoxin-producing cultures of *Aspergillus flavus*. Tetrahedron Lett..

[57] Pubchem (2020). Explore Chemistry. https://pubchem.ncbi.nlm.nih.gov.

[58] Chemspider (2020). Search and Share Chemistry. http://www.chemspider.com.

[59] Lai D.Y., Woo Y.T., Arcos J.C., Argus M.F. (1985). Difuroxanthone-, Difurocoumarolactone- and Difuroanthraquinone-Type Alkylating Agents: Carcinogenicity and Structure Activity Relationships: Other Biological Properties: Metabolism: Environmental Significance. https://www.epa.gov/nscep.

[60] Dutton M.F., Heathcote J.G. (1968). The structure, biochemical properties and origin of the aflatoxins B2a and G2a. Chem. Ind..

[61] Lillehoj E.B., Ciegler A. (1969). Biological activity of aflatoxin B2a. Appl. Microbiol..

[62] IARC (International Agency for Research on Cancer) (2012). Chemical agents and related occupations. IARC Monographs on the Evaluation of Carcinogenic Risks to Humans.

[63] Bbosa G., Kitya D., Odda J., Ogwal-Okeng J. (2013). Aflatoxins metabolism, effects on epigenetic mechanisms and their role in carcinogenesis. Health (N. Y.).

[64] Rodricks J.V., Henery-Logan K.R., Campbell A.D., Stoloff L., Verrett M.J. (1968). Isolation of a New Toxin from Cultures of Aspergillus flavus. Nature.

[65] Wood G.E. (1992). Mycotoxins in foods and feeds in the United States. J. Anim. Sci..

[66] Wu F., Narrod C., Tiongco M., Liu Y. (2011). The Health Economics of Aflatoxin: Global Burden of Disease. http://www.ifpri.org/publication/health-economics-aflatoxin.

[67] Cotty P.J., Jaime-Garcia R. (2007). Influences of climate on aflatoxin producing fungi and aflatoxin contamination. Int. J. Food Microbiol..

[68] Horn B.W., Dorner J.W. (2002). Effect of competition and adverse culture conditions on aflatoxin production by Aspergillus flavus through successive generations. Mycologia.

[69] Smith L.E., Stasiewicz M.J., Hestrin R., Morales L., Mutiga S., Nelson R.J. (2016). Examining environmental drivers of spatial variability in aflatoxin accumulation in Kenyan maize: Potential utility in risk prediction models. Afr. J. Food Agric. Nutr. Dev..

[70] Abdel-Hadi A., Schmidt-Heydt M., Parra R., Geisen R., Magan N. (2012). A systems approach to model the relationship between aflatoxin gene cluster expression, environmental factors, growth and toxin production by *Aspergillus flavus*. J. Royal Society Interface.

[71] Sirma A.J., Lindahl J.F., Makita K., Senerwa D., Mtimet N., Kang’ethe E.K., Grace D. (2018). The impacts of aflatoxin standards on health and nutrition in sub-Saharan Africa: The case of Kenya. Glob. Food Secur..

[72] Anukul N., Vangnai K., Mahakarnchanakul W. (2013). Significance of regulation limits in mycotoxin contamination in Asia and risk management programs at the national level. J. Food Drug Anal..

[73] Kottek M., Grieser J., Beck C., Rudolf B., Rubel F. (2006). World Map of the Köppen-Geiger climate classification updated. Meteorolog. Z..

[74] Kaaya A.N., Warren H.L., Kyamanywa S., Kyamuhangire W. (2005). The effect of delayed harvest on moisture content, insect damage, moulds and aflatoxin contamination of maize in Mayuge district of Uganda. J. Sci. Food Agric..

[75] Iqbal M., Abbas M., Adil M., Nazir A., Ahmad I. (2019). Aflatoxins biosynthesis, toxicity and intervention strategies: A review. Chem. Int..

[76] Dawd Gashu D., Demment M.W., Stoecker B.J. (2019). Challenges and opportunities to the African agriculture and food systems. Afr. J. Food Agric. Nutr. Dev..

[77] Njoroge A.W., Baoua I., Baributsa D. (2019). Postharvest management practices of grains in the Eastern region of Kenya. J. Agric. Sci..

[78] Kagot V., Okoth S., De Boevre M., De Saeger S. (2019). Biocontrol of Aspergillus and Fusarium Mycotoxins in Africa: Benefits and Limitations. Toxins (Basel).

[79] Lukwago F.B., Mukisa I.M., Atukwase A., Kaaya A.N., Tumwebaze S. (2019). Mycotoxins contamination in foods consumed in Uganda: A 12-year review (2006–18). Sci. Afr..

[80] Ingenbleek L., Sulyok M., Adegboye A., Hossou E.S., Koné Z.A., Oyedele D.A., Kisito K.J., Sika C., Koreissi Dembélé Y., Eyangoh S. (2019). Regional Sub-Saharan Africa Total Diet Study in Benin, Cameroon, Mali and Nigeria Reveals the Presence of 164 Mycotoxins and Other Secondary Metabolites in Foods. Toxins (Basel).

[81] Do T.H., Tran S.C., Le C.D., Nguyen H.-B.T., Le P.-T.T., Le H.-H.T., Le T.D., Thai-Nguyen H.-T. (2020). Dietary exposure and health risk characterization of aflatoxin B1, ochratoxin A, fumonisin B1, and zearalenone in food from different provinces in Northern Vietnam. Food Control.

[82] Hussaini A.M., Timothy A.G., Olufunmilayo H.A., Ezekiel A.S., Godwin H.O. (2009). Fungi and some mycotoxins found in mouldy Sorghum in Niger State, Nigeria. World J. Agric. Sci..

[83] Okoth S., Kola M. (2012). Market samples as a source of chronic aflatoxin exposure in Kenya. Afr. J. Health Sci..

[84] Lewis L., Onsongo M., Njapau H., Schurz-Rogers H., Luber G., Kieszak S., Nyamongo J., Backer L., Dahiye A.M., Misore A. (2005). Aflatoxin contamination of commercial maize products during an outbreak of acute aflatoxicosis in eastern and central Kenya. Environ. Health Perspect..

[85] Ngindu A., Kenya P., Ocheng D., Omondi T., Ngare W., Gatei D., Johnson B., Ngira J., Nandwa H., Jansen A. (1982). Outbreak of acute hepatitis caused by aflatoxin poisoning in Kenya. Lancet.

[86] Mahuku G., Nzioki H.S., Mutegi C., Kanampiu F., Narrod C., Makumbi D. (2019). Pre-harvest management is a critical practice for minimizing aflatoxin contamination of maize. Food Control.

[87] Afsah-Hejri L., Jinap S., Hajeb P., Radu S., Shakibazadeh S. (2013). A Review on Mycotoxins in Food and Feed: Malaysia Case Study. Compr. Rev. Food Sci. Food Safety.

[88] Kuiper-Goodman T. (1995). Mycotoxins: Risk assessment and legislation. Toxicol. Lett..

[89] Odera J.O., Odera E., Githang’a J., Walong E.O., Li F., Xiong Z., Chen X.L. (2017). Esophageal cancer in Kenya. Am. J. Dig. Dis..

[90] Sserumaga J.P., Ortega-Beltran A., Wagacha J.M., Mutegi C.K., Bandyopadhyay R. (2020). Aflatoxin-producing fungi associated with pre-harvest maize contamination in Uganda. Int. J. Food Microbiol..

[91] Hamidou F., Rathore A., Waliyar F., Vadez V. (2014). Although drought intensity increases aflatoxin contamination, drought tolerance does not lead to less aflatoxin contamination. Field Crop. Res..

[92] Cardwell K.F., Henry S.H. (2004). Risk of exposure to and mitigation of effect of aflatoxin on human health: A West African example. J. Toxicol. Toxin Rev..

[93] Hell K., Cardwell K.F., Setamou M., Poehling H.M. (2000). The influence of storage practices on aflatoxin contamination in maize in four agroecological zones of Benin, west Africa. J. Stored Prod. Res..

[94] Udoh J.M., Cardwell K.F., Ikotun T. (2000). Storage structures and aflatoxin content of maize in five agroecological zones of Nigeria. J. Stored Prod. Res..

[95] Ngoko Z., Marasas W.F.O., Rheeder J.P., Shephard G.S., Wingfield M.J., Cardwell K.F. (2001). Fungal infection and mycotoxin contamination of maize in the Humid forest and the western highlands of Cameroon. Phytoparasitica.

[96] Hell K., Cardwell K.F., Setamou M., Schulthess F. (2000). Influence of insect infestation on aflatoxin contamination of stored maize in four agroecological regions in Benin. Afr. Entomol..

[97] Cardwell K.F. (2000). Mycotoxin Contamination of foods in Africa: Antinutritional factors. Food Nutr. Bull..

[98] Torres A.M., Barros G.G., Palacios S.A., Chulze S.N., Battilani P. (2014). Review on pre- and post-harvest management of peanuts to minimize aflatoxin contamination. Food Res. Int..

[99] Marete N.G., Kanja W.L., Mbaria M.J., Okumu O.M., Ateku A.P., Korhonen H., Joutsjoki V. (2019). Effects of the Use of good agricultural practices on aflatoxin levels in maize grown in Nandi county, Kenya. Science.

[100] Seetha A., Munthali W., Msere H.W., Swai E., Muzanila Y., Sichone E., Tsusaka T.W., Rathore A., Okori P. (2017). Occurrence of aflatoxins and its management in diverse cropping systems of central Tanzania. Mycotoxin Res..

[101] World Bank Group (2019). Climate Change Knowledge Portal. https://climateknowledgeportal.worldbank.org.

[102] (2019). Climatedata.eu. Climate. https://www.climatedata.eu/index.php?lang=en.

[103] Institute for Veterinary Public Health World Maps of KöPpen-Geiger Climate Classification. http://koeppen-geiger.vu-wien.ac.at.

[104] Niles E.V., Norman J.A., Pimbley D. (1985). Growth and aflatoxin production of Aspergillus flavus in wheat and barley. Trans. Br. Mycol. Soc..

[105] Gizachew D., Chang C.-H., Szonyi B., De La Torre S., Ting W.-t.E. (2019). Aflatoxin B1 (AFB1) production by *Aspergillus flavus* and *Aspergillus parasiticus* on ground Nyjer seeds: The effect of water activity and temperature. Int. J. Food Microbiol..

[106] Lahouar A., Marin S., Crespo-Sempere A., Saïd S., Sanchis V. (2016). Effects of temperature, water activity and incubation time on fungal growth and aflatoxin B1 production by toxinogenic Aspergillus flavus isolates on sorghum seeds. Rev. Argent. Microbiol..

[107] Lv C., Jin J., Wang P., Dai X., Liu Y., Zheng M., Xing F. (2019). Interaction of water activity and temperature on the growth, gene expression and aflatoxin production by Aspergillus flavus on paddy and polished rice. Food Chem..

[108] Holmquist G.U., Walker H.W., Stahr H.M. (1983). Influence of Temperature, pH, Water Activity and Antifungal Agents on Growth of *Aspergillus flavus* and A. parasiticus. J. Food Sci..

[109] Schmidt-Heydt M., Rüfer C.E., Abdel-Hadi A., Magan N., Geisen R. (2010). The production of aflatoxin B1 or G1 by Aspergillus parasiticus at various combinations of temperature and water activity is related to the ratio of aflS to aflR expression. Mycotoxin Res..

[110] Northolt M.D., van Egmond H.P., Paulsch W.E. (1977). Differences Between Aspergillus flavus Strains in Growth and Aflatoxin B1 Production in Relation to Water Activity and Temperature. J. Food Prot..

[111] Northolt M.D., Verhulsdonk C.A.H., Soentoro P.S.S., Paulsch W.E. (1976). Effect of Water Activity and Temperature on Aflatoxin Production by Aspergillus parasiticus. J. Milk Food Technol..

[112] Tennigkeit T., Vincent K. (2014). Adaptation of Agricultural Practices to Climate Change in Sub-Saharan Africa (CAADP). Good Agricultural Adaptation Practices: South Africa. http://kulima.com/wp-content/uploads/2015/12/CAADP-South-African-Final-Report.pdf.

[113] Meyer H., Skhosana D.Z., Motlanthe M., Louw W., Rohwer E. (2019). Long term monitoring (2014–2018) of multi-mycotoxins in South African commercial maize and wheat with a locally developed and validated LC-MS/MS method. Toxins (Basel).

[114] Medina A., Rodriguez A., Magan N. (2014). Effect of climate change on *Aspergillus flavus* and aflatoxin B1 production. Front. Microbiol..

[115] Anyamba A., Chretien J.-P., Britch S.C., Soebiyanto R.P., Small J.L., Jepsen R., Forshey B.M., Sanchez J.L., Smith R.D., Harris R. (2019). Global disease outbreaks associated with the 2015–2016 El Niño event. Sci. Rep..

[116] Krishnamachari K.A.V.R., Nagarajan V., Bhat R., Tilak T.B.G. (1975). Hepatitis due to aflatoxicosis: An outbreak in Western India. Lancet.

[117] Villers P. (2017). Food safety and aflatoxin control. J. Food Res..

[118] PDNA (post-disaster needs assessment) (2012). Kenya Post-disaster Needs Assessment 2008–2011 Drought Report: 2012 European Union/United Nations: Government of Kenya. World Bank. http://www.gfdrr.org/sites/gfdrr/files/Kenya_PDNA_Final.

[119] Shank R.C., Gordon J.E., Wogan G.N., Nondasuta A., Subhamani B. (1972). Dietary aflatoxins and human liver cancer. III. Field survey of rural Thai families for ingested aflatoxins. Food Cosmet. Toxicol..

[120] Shank R.C., Wogan G.N., Gibson J.B., Nondasuta A. (1972). Dietary aflatoxins and human liver cancer. II. Aflatoxins in market foods and foodstuffs of Thailand and Hong Kong. Food Cosmet. Toxicol..

[121] Liverpool-Tasie L.S.O., Turna N.S., Ademola O., Obadina A., Wu F. (2019). The occurrence and co-occurrence of aflatoxin and fumonisin along the maize value chain in southwest Nigeria. Food Chem. Toxicol..

[122] Mutegi C., Wagacha M., Kimani J., Otieno G., Wanyama R., Hell K., Christie M.E. (2013). Incidence of aflatoxin in peanuts (*Arachis hypogaea* Linnaeus) from markets in Western, Nyanza and Nairobi Provinces of Kenya and related market traits. J. Stored Prod. Res..

[123] Gruber-Dorninger C., Jenkins T., Schatzmayr G. (2019). Global mycotoxin occurrence in feed: A ten-year survey. Toxins (Basel).

[124] Kitya D., Bbosa G.S., Mulogo E. (2010). Aflatoxin levels in common foods of South Western Uganda: A risk factor to hepatocellular carcinoma. Eur. J. Cancer Care.

[125] Baluka S.A., Schrunk D., Imerman P., Kateregga J.N., Camana E., Wang C., Rumbeiha W.K. (2017). Mycotoxin and metallic element concentrations in peanut products sold in Ugandan markets. Cogent Food & Agric..

[126] Sirma A.J., Senerwa D.M., Grace D., Makita K., Mtimet N., Kang’ethe E.K., Lindahl J.F. (2016). Aflatoxin B1 occurrence in millet, sorghum and maize from four agro-ecological zones in Kenya. Afr. J. Food Agric. Nutr. Dev..

[127] Menza N., Muturi M., Kamau M.L. (2015). Incidence, types and levels of aflatoxin in different peanuts varietiesproduced in Busia and Kisii central districts, Kenya. Open J. Med. Microbiol..

[128] Nyandieka H.S., Nyamogoba H.D., Nyamwange C.I. (2014). Distribution of aflatoxins and micro organisms in peanut and sunflower seed products and their potential health hazards. Pak. J. Med. Res..

[129] Kang’ethe E.K., Sirma A.J., Murithi G., Mburugu-Mosoti C.K., Ouko E.O., Korhonen H.J., Nduhiu G.J., Mungatu J.K., Joutsjoki V., Lindfors E. (2017). Occurrence of mycotoxins in food, feed, and milk in two counties from different agro-ecological zones and with historical outbreak of aflatoxins and fumonisins poisonings in Kenya. Food Qual. Saf..

[130] Gachara G.W., Nyamache A.K., Harvey J., Gnonlonfin G.J.B., Wainaina J. (2018). Genetic diversity of *Aspergillus flavus* and occurrence of aflatoxin contamination in stored maize across three agro-ecological zones in Kenya. Agri. & Food Secur..

[131] Daniel J.H., Lewis L.W., Redwood Y.A., Kieszak S., Breiman R.F., Flanders W.D., Bell C., Mwihia J., Ogana G., Likimani S. (2011). Comprehensive assessment of maize aflatoxin levels in Eastern Kenya, 2005–2007. Environ. Health Perspect..

[132] Kiarie G., Dominguez-Salas P., Kang’Ethe S., Grace D., Lindahl J. (2016). Aflatoxin exposure among young children in urban low-income areas of Nairobi and association with child growth. Afr. J. Food Agr. Nutr. Dev..

[133] Kang’ethe E., Gatwiri M., Sirma A., Ouko E., Mburugu-Musoti C., Kitala P., Nduhiu G., Nderitu J., Mungatu J., Hietaniemi V. (2017). Exposure of Kenyan population to aflatoxins in foods with special reference to Nandi and Makueni counties. Food Qual. Saf..

[134] Nyangi C., Beed F., Mugula J., Boni S., Koyano E., Mahuku G., Sulyok M., Bekunda M. (2016). Assessment of pre-harvest aflatoxin and fumonisin contamination of maize in Babati District, Tanzania. Afr. J. Food Agric. Nutr. Dev..

[135] Kimanya M.E., De Meulenaer B., Tiisekwa B., Ndomondo-Sigonda M., Devlieghere F., Van Camp J., Kolsteren P. (2008). Co-occurrence of fumonisins with aflatoxins in home-stored maize for human consumption in rural villages of Tanzania. Food Addit. Contam. Part A.

[136] Kamala A., Ortiz J., Kimanya M., Haesaert G., Donoso S., Tiisekwa B., De Meulenaer B. (2015). Multiple mycotoxin co-occurrence in maize grown in three agro-ecological zones of Tanzania. Food Control.

[137] Mmongoyo J.A., Wu F., Linz J.E., Nair M.G., Mugula J.K., Tempelman R.J., Strasburg G.M. (2017). Aflatoxin levels in sunflower seeds and cakes collected from micro- and small-scale sunflower oil processors in Tanzania. PLoS ONE.

[138] Mngqawa P., Shephard G.S., Green I.R., Ngobeni S.H., de Rijk T.C., Katerere D.R. (2016). Mycotoxin contamination of home-grown maize in rural northern South Africa (Limpopo and Mpumalanga Provinces). Food Addit. Contam. Part B.

[139] Kamika I., Mngqawa P., Rheeder J.P., Teffo S.L., Katerere D.R. (2014). Mycological and aflatoxin contamination of peanuts sold at markets in Kinshasa, Democratic Republic of Congo, and Pretoria, South Africa. Food Addit. Contam. Part B.

[140] Salau I.A., Shehu K., Muhammad S., Umar R.A. (2016). Aflatoxin contamination of stored groundnut kernel in Sokoto State, Nigeria. Greener J. Agric. Sci..

[141] Oloyede M., Williams A., Benson N. (2016). Aflatoxin Contamination of Some Edible Grains from Lagos and Ota Markets, Nigeria. Environ. Sci. Technol..

[142] Adebajo L.O., Idowu A.A., Adesanya O.O. (1994). Mycoflora, and mycotoxins production in Nigerian corn and corn-based snacks. Mycopathologia.

[143] Njumbe Ediage E., Hell K., De Saeger S. (2014). A comprehensive study to explore differences in mycotoxin patterns from agro-ecological regions through maize, peanut, and cassava products: A case study, Cameroon. J. Agric. Food Chem..

[144] Agbetiameh D., Ortega-Beltran A., Awuah R.T., Atehnkeng J., Cotty P.J., Bandyopadhyay R. (2017). Prevalence of Aflatoxin Contamination in Maize and Groundnut in Ghana: Population Structure, Distribution, and Toxigenicity of the Causal Agents. Plant Dis..

[145] Hanvi D.M., Lawson-Evi P., De Boevre M., Goto C.E., De Saeger S., Eklu-Gadegbeku K. (2019). Natural occurrence of mycotoxins in maize and sorghum in Togo. Mycotoxin Res..

[146] Dadzie M.A., Oppong A., Ofori K., Eleblu J.S., Ifie E.B., Blay E., Obeng –Bio E., Appiah-Kubi Z., Warburton M.L. (2019). Distribution of Aspergillus flavus and aflatoxin accumulation in stored maize grains across three agro-ecologies in Ghana. Food Control.

[147] Van Rensburg S.J., Cook-Mozaffari P., Van Schalkwyk D.J., Van der Watt J.J., Vincent T.J., Purchase I.F. (1985). Hepatocellular carcinoma and dietary aflatoxin in Mozambique and Transkei. Br. J. Cancer.

[148] Wyk P.S.v., Merwe P.J.A.V.d., Subrahmanyam P., Boughton D. (1999). Aflatoxin contamination of groundnuts in Mozambique. Int. Arachis Newsl..

[149] Cambaza E., Koseki S., Kawamura S. (2018). Aflatoxins in Mozambique: Etiology, Epidemiology and Control. Agriculture.

[150] Reddy K.R., Reddy C.S., Muralidharan K. (2009). Detection of *Aspergillus* spp. and aflatoxin B1 in rice in India. Food Microbiol..

[151] Mohana D., Thippeswamy S., Abhishek R., Shobha B., Mamatha M. (2017). Studies on seed-borne mycoflora and aflatoxin B1 contaminations in food based seed samples: Molecular detection of mycotoxigenic Aspergillus flavus and their management. Int. Food Res. J..

[152] Bhat R.V., Vasanthi S., Rao B.S., Rao R.N., Rao V.S., Nagaraja K.V., Bai R.G., Prasad C.A.K., Vanchinathan S., Roy R. (1997). Aflatoxin b1 contamination in maize samples collected from different geographical regions of India—a multicentre study. Food Addit. Contam. Part B.

[153] Ratnavathi C.V., Komala V.V., Chavan U.D., Ratnavathi C.V., Patil J.V., Chavan U.D. (2016). Chapter 3—Mycotoxin Contamination in Sorghum. Sorghum Biochemistry.

[154] Siruguri V., Kumar P.U., Raghu P., Rao M.V., Sesikeran B., Toteja G.S., Gupta P., Rao S., Satyanarayana K., Katoch V.M. (2012). Aflatoxin contamination in stored rice variety PAU 201 collected from Punjab, India. Indian J. Med. Res..

[155] Koirala P., Kumar S., Yadav B.K., Premarajan K.C. (2005). Occurrence of aflatoxin in some of the food and feed in Nepal. Indian J. Med. Sci..

[156] Quitco R.T., Champ B.R., Highley E., Hocking A.D., Pitt J.I. (1991). Aflatoxin studies in the Philippines. Fungi and mycotoxins in stored products, Proceedings of an international conference held in Bankok, Thailand, 23–26 April 1991.

[157] Arim R.H. (2004). Mycotoxin contamination of food and feeds in the Philippines. JSM Mycotoxins.

[158] Sales A.C., Yoshizawa T. (2005). Updated profile of aflatoxin and Aspergillus section Flavi contamination in rice and its byproducts from the Philippines. Food Addit. Contam. Part B.

[159] Panrapee I., Phakpoom K., Thanapoom M., Nampeung A., Warapa M. (2016). Exposure to aflatoxin B1 in Thailand by consumption of brown and color rice. Mycotoxin Res..

[160] Kooprasertying P., Maneeboon T., Hongprayoon R., Mahakarnchanakul W. (2016). Exposure assessment of aflatoxins in Thai peanut consumption. Cogent Food & Agric..

[161] Leong Y.-H., Ismail N., Latif A.A., Ahmad R. (2010). Aflatoxin occurrence in nuts and commercial nutty products in Malaysia. Food Control.

[162] Khayoon W.S., Saad B., Lee T.P., Salleh B. (2012). High performance liquid chromatographic determination of aflatoxins in chilli, peanut and rice using silica based monolithic column. Food Chem..

[163] Semple R.L., Frio A.S., Hicks P.A., Lozare J.V. (1991). Mycotoxin Prevention and Control in Foodgrains. http://www.fao.org/3/X5036E/x5036E1b.htm#Mycotoxins%20in%20foodgrains%20in%20some%20Asian%20countries.

[164] Arzandeh S., Selamat J., Lioe H. (2010). Aflatoxin in raw peanut kernels Marketed in Malaysia. J. Food Drug Anal..

[165] Soleimany F., Jinap S., Faridah A., Khatib A. (2012). A UPLC–MS/MS for simultaneous determination of aflatoxins, ochratoxin A, zearalenone, DON, fumonisins, T-2 toxin and HT-2 toxin, in cereals. Food Control.

[166] Reddy K.R., Farhana N.I., Salleh B. (2011). Occurrence of *Aspergillus* spp. and aflatoxin B1 in Malaysian foods used for human consumption. J. Food Sci..

[167] Frederick W.H., Leinbach T.R. Southeast Asia 2018: Encyclopædia Britannica, Inc.. https://www.britannica.com/place/Southeast-Asia.

[168] Reddy B.N., Raghvender C.N. (2007). Outbreaks of aflatoxicosis in India. Afr. J. Food Agric. Nutr. Dev..

[169] Hamid A.S., Tesfamariam I.G., Zhang Y., Zhang Z.G. (2013). Aflatoxin B1-induced hepatocellular carcinoma in developing countries: Geographical distribution, mechanism of action and prevention. Oncol. Lett..

[170] Tolosa J., Rodríguez-Carrasco Y., Ferrer E., Mañes J. (2019). Identification and Quantification of Enniatins and Beauvericin in Animal Feeds and Their Ingredients by LC-QTRAP/MS/MS. Metabolites.

[171] Senerwa D. (2016). Prevalence of aflatoxin in feeds and cow milk from five counties in Kenya. Afr. J. Food Agric. Nutr. Dev..

[172] Borutova R., Aragon Y.A., Nährer K., Berthiller F. (2012). Co-occurrence and statistical correlations between mycotoxins in feedstuffs collected in the Asia–Oceania in 2010. Anim. Feed Sci. Tech..

[173] Anjum M.A., Khan S.H., Sahota A.W., Sardar R. (2012). Assessment of Aflatoxin B1 in commercial poultry feed and feed ingredients. J. Anim. Plant Sci..

[174] Tangendjaja B., Rachmawati S., Wina E. (2008). Mycotoxin contamination on corn used by feed mills in indonesia. Indones. J. Agric. Sci..

[175] Sarma U.P., Bhetaria P.J., Devi P., Varma A. (2017). Aflatoxins: Implications on health. Indian J. Clin. Biochem..

[176] Dilkin P., Zorzete P., Mallmann C.A., Gomes J.D., Utiyama C.E., Oetting L.L., Correa B. (2003). Toxicological effects of chronic low doses of aflatoxin B(1) and fumonisin B(1)-containing *Fusarium moniliforme* culture material in weaned piglets. Food Chem. Toxicol..

[177] WHO (World Health Organization) (2017). Evaluation of certain contaminants in food: eighty-third report of the Joint FAO/WHO Expert Committee on Food Additives.

[178] Salhab A.S., Edwards G.S. (1977). Comparative in vitro metabolism of aflatoxicol by liver preparations from animals and humans. Cancer Res..

[179] Smith J.W., Groopman J.D., Boffetta P., Hainaut P. (2019). Aflatoxins. Encyclopedia of Cancer (Third Edition).

[180] Peraica M., Radić B., Lucić A., Pavlović M. (1999). Toxic effects of mycotoxins in humans. Bull. World Health Organ..

[181] Williams J.H., Phillips T.D., Jolly P.E., Stiles J.K., Jolly C.M., Aggarwal D. (2004). Human aflatoxicosis in developing countries: A review of toxicology, exposure, potential health consequences, and interventions. Am. J. Clin. Nutr..

[182] Chao T.C., Maxwell S.M., Wong S.Y. (1991). An outbreak of aflatoxicosis and boric acid poisoning in Malaysia: A clinicopathological study. J. Pathol..

[183] Kamala A., Shirima C., Jani B., Bakari M., Sillo H., Rusibamayila N., De Saeger S., Kimanya M., Gong Y.Y., Simba A. (2018). Outbreak of an acute aflatoxicosis in Tanzania during 2016. World Mycotoxin J..

[184] Serck-Hanssen A. (1970). Aflatoxin-induced fatal hepatitis? A case report from Uganda. Arch. Environ. Health.

[185] Lye M.S., Ghazali A.A., Mohan J., Alwin N., Nair R.C. (1995). An outbreak of acute hepatic encephalopathy due to severe aflatoxicosis in Malaysia. Am. J. Trop. Med. Hyg..

[186] Willis R., Mulvihill J., Hoofnagle J. (1980). Attempted suicide with purified aflatoxin. Lancet.

[187] Mupunga I., Mngqawa P., Katerere D.R. (2017). Peanuts, aflatoxins and undernutrition in children in sub-saharan Africa. Nutrients.

[188] Marijani E., Nasimolo J., Kigadye E., Gnonlonfin G.J.B., Okoth S. (2017). Sex-related differences in hematological parameters and organosomatic indices of oreochromis niloticus exposed to aflatoxin B diet. Scientifica.

[189] Monson S.M., Coulombe A.R., Reed M.K. (2015). Aflatoxicosis: Lessons from toxicity and responses to aflatoxin B1 in poultry. Agriculture.

[190] Ishikawa A.T., Hirooka E.Y., Alvares E Silva P.L., Bracarense A.P.F.R.L., Flaiban K.K.M.d.C., Akagi C.Y., Kawamura O., Costa M.C.d., Itano E.N. (2017). Impact of a single oral acute dose of aflatoxin B₁ on liver function/cytokines and the lymphoproliferative response in C57Bl/6 mice. Toxins (Basel).

[191] Diaz G.J., Murcia H.W. (2019). An unusually high production of hepatic aflatoxin B1-dihydrodiol, the possible explanation for the high susceptibility of ducks to aflatoxin B1. Sci. Rep..

[192] Anon (1976). Epidemic of hepatitis in man due to aflatoxicosis. Nutr. Rev..

[193] CDC (Centers for Disease Control and Prevention) (2004). Outbreak of aflatoxin poisoning--eastern and central provinces, Kenya, January-July 2004. MMWR Morb. Mortal. Wkly. Rep..

[194] Outbreak News Today (2017). Aflatoxin Kills 4 Children in Tanzania, Linked to Consumption of Maize. http://outbreaknewstoday.com.

[195] Azziz-Baumgartner E., Lindblade K., Gieseker K., Rogers H.S., Kieszak S., Njapau H., Schleicher R., McCoy L.F., Misore A., DeCock K. (2005). Case-control study of an acute aflatoxicosis outbreak, Kenya, 2004. Environ. Health Perspect..

[196] Probst C., Njapau H., Cotty P.J. (2007). Outbreak of an acute aflatoxicosis in Kenya in 2004: Identification of the causal agent. Appl. Environ. Microbiol..

[197] Infopedia (2019). Nine Emperor Gods Festival. https://eresources.nlb.gov.sg/infopedia/articles/SIP_1849_2011–10-21.html.

[198] Qian G., Tang L., Lin S., Xue K.S., Mitchell N.J., Su J., Gelderblom W.C., Riley R.T., Phillips T.D., Wang J.-S. (2016). Sequential dietary exposure to aflatoxin B1 and fumonisin B1 in F344 rats increases liver preneoplastic changes indicative of a synergistic interaction. Food Chem. Toxicol..

[199] WHO (World Health Organization) (2017). Weekly bulletin on outbreaks and other emergencies: Week 32: 19–25 August 2017. http://www.who.int/iris/handle/10665/258794.

[200] Food and Agriculture Organization of the United Nations, Expert Committee on Food Additives Meeting World Health Organization International Programme on Chemical, Safety. Safety Evaluation of Certain Food Additives and Contaminants, prepared by the Forty-Ninth Meeting of the Joint FAO/WHO Expert Committee on Food Additives (JEFCA)1998.

[201] Shephard G.S. (2008). Risk assessment of aflatoxins in food in Africa. Food Addit. Contam. Part A.

[202] Bowers J., Brown B., Springer J., Tollefson L., Lorentzen R., Henry S. (1993). Risk assessment for aflatoxin: An evaluation based on the multistage model. Risk Anal..

[203] EFSA (European Food Safety Authority) (2005). Opinion of the Scientific Committee on a request from EFSA related to a harmonised approach for risk assessment of substances which are both genotoxic and carcinogenic. EFSA J..

[204] Wu F., Stacy S.L., Kensler T.W. (2013). Global risk assessment of aflatoxins in maize and peanuts: Are regulatory standards adequately protective?. Toxicol. Sci..

[205] WHO (World Health organization) (2006). Global Environment Monitoring System-food Contamination Monitoring and Assessment Programme (GEMS/Food). http://www.who.int/foodsafety/chem/gems/en/index1.html.2006.

[206] Johnson N.M., Egner P.A., Baxter V.K., Sporn M.B., Wible R.S., Sutter T.R., Groopman J.D., Kensler T.W., Roebuck B.D. (2014). Complete protection against aflatoxin B(1)-induced liver cancer with a triterpenoid: DNA adduct dosimetry, molecular signature, and genotoxicity threshold. Cancer Prev. Res. (Phila.).

[207] Gan L.S., Skipper P.L., Peng X.C., Groopman J.D., Chen J.S., Wogan G.N., Tannenbaum S.R. (1988). Serum albumin adducts in the molecular epidemiology of aflatoxin carcinogenesis: Correlation with aflatoxin B1 intake and urinary excretion of aflatoxin M1. Carcinogenesis.

[208] Wild C.P., Hasegawa R., Barraud L., Chutimataewin S., Chapot B., Ito N., Montesano R. (1996). Aflatoxin-albumin adducts: A basis for comparative carcinogenesis between animals and humans. Cancer Epidemiol. Biomark. Prev..

[209] Gong Y.Y., Hounsa A., Egal S., Turner P.C., Sutcliffe A.E., Hall A.J., Cardwell K., Wild C.P. (2004). Postweaning exposure to aflatoxin results in impaired child growth: a longitudinal study in Benin, West Africa. Environ. Health Perspect..

[210] Gong Y.Y., Egal S., Hounsa A., Turner P.C., Hall A.J., Cardwell K.F., Wild C.P. (2003). Determinants of aflatoxin exposure in young children from Benin and Togo, West Africa: The critical role of weaning. Int. J. Epidemiol..

[211] Gong Y.Y., Cardwell K., Hounsa A., Egal S., Turner P.C., Hall A.J., Wild C.P. (2002). Dietary aflatoxin exposure and impaired growth in young children from Benin and Togo: Cross sectional study. BMJ.

[212] Hojnik N., Cvelbar U., Tavčar-Kalcher G., Walsh J.L., Križaj I. (2017). Mycotoxin decontamination of food: Cold atmospheric pressure plasma versus “classic” decontamination. Toxins (Basel).

[213] Chen R., Ma F., Li P.-W., Zhang W., Ding X.-X., Zhang Q., Li M., Wang Y.-R., Xu B.-C. (2014). Effect of ozone on aflatoxins detoxification and nutritional quality of peanuts. Food Chem..

[214] Luo X., Wang R., Wang L., Li Y., Bian Y., Chen Z. (2014). Effect of ozone treatment on aflatoxin B1 and safety evaluation of ozonized corn. Food Control.

[215] Agriopoulou S., Koliadima A., Karaiskakis G., Kapolos J. (2016). Kinetic study of aflatoxins’ degradation in the presence of ozone. Food Control.

[216] Porto D.Y., Trombete M.F., Freitas-Silva O., de Castro M.I., Direito M.G., Ascheri L.J. (2019). Gaseous ozonation to reduce aflatoxins levels and microbial contamination in corn grits. Microorganisms.

[217] Rushing B.R., Selim M.I. (2019). Aflatoxin B1: A review on metabolism, toxicity, occurrence in food, occupational exposure, and detoxification methods. Food Chem. Toxicol..

[218] Jard G., Liboz T., Mathieu F., Guyonvarc’h A., Lebrihi A. (2011). Review of mycotoxin reduction in food and feed: From prevention in the field to detoxification by adsorption or transformation. Food Addit. Contam. Part A.

[219] Jardon-Xicotencatl S., Díaz-Torres R., Marroquín-Cardona A., Villarreal-Barajas T., Méndez-Albores A. (2015). Detoxification of aflatoxin-contaminated maize by neutral electrolyzed oxidizing water. Toxins (Basel).

[220] Udomkun P., Wiredu A.N., Nagle M., Müller J., Vanlauwe B., Bandyopadhyay R. (2017). Innovative technologies to manage aflatoxins in foods and feeds and the profitability of application—A review. Food Control.

[221] Peng Z., Chen L., Zhu Y., Huang Y., Hu X., Wu Q., Nüssler A.K., Liu L., Yang W. (2018). Current major degradation methods for aflatoxins: A review. Trends Food. Sci. Technol..

[222] Branà M.T., Cimmarusti M.T., Haidukowski M., Logrieco A.F., Altomare C. (2017). Bioremediation of aflatoxin B1-contaminated maize by king oyster mushroom (Pleurotus eryngii). PLoS ONE.

[223] Kim S., Lee H., Lee S., Lee J., Ha J., Choi Y., Yoon Y., Choi K.-H. (2017). Invited review: Microbe-mediated aflatoxin decontamination of dairy products and feeds. J. Dairy Sci..

[224] Bond J.Q., Jungong C.S., Chatzidimitriou A. (2016). Microkinetic analysis of ring opening and decarboxylation of γ-valerolactone over silica alumina. J. Catal..

[225] Benkerroum N. (2020). Chronic and Acute Toxicities of Aflatoxins: Mechanisms of Action. Int. J. Environ. Res. Public Health.

[226] Iram W., Anjum T., Iqbal M., Ghaffar A., Abbas M., Khan A.M. (2016). Structural analysis and biological toxicity of aflatoxins B1 and B2 degradation products following detoxification by *Ocimum basilicum* and cassia fistula aqueous extracts. Front. Microbiol..

[227] Lapalikar G.V., Taylor M.C., Warden A.C., Scott C., Russell R.J., Oakeshott J.G. (2012). F420H2-dependent degradation of aflatoxin and other furanocoumarins is widespread throughout the actinomycetales. PLoS ONE.

[228] Lyagin I., Efremenko E. (2019). Enzymes for detoxification of various mycotoxins: Origins and mechanisms of catalytic action. Molecules.

[229] USEPA (2003). Biopesticide registration action document *Aspergillus flavus* AF36. https://www3.epa.gov/pesticides/chem_search/reg_actions/registration/decision_PC-006456_3-Jul-03.pdf.

[230] Dorner J.W. (2009). Development of Biocontrol Technology to Manage Aflatoxin Contamination in Peanuts. Peanut Sci..

[231] Agbetiameh D., Ortega-Beltran A., Awuah R.T., Atehnkeng J., Islam M.-S., Callicott K.A., Cotty P.J., Bandyopadhyay R. (2019). Potential of atoxigenic *Aspergillus flavus* vegetative compatibility groups associated with maize and groundnut in Ghana as biocontrol agents for aflatoxin management. Front. Microbiol..

[232] Mauro A., Garcia-Cela E., Pietri A., Cotty J.P., Battilani P. (2018). Biological control products for aflatoxin prevention in Italy: Commercial field evaluation of atoxigenic *Aspergillus flavus* active ingredients. Toxins (Basel).

[233] Bandyopadhyay R., Atehnkeng J., Ortega-Beltran A., Akande A., Falade T.D.O., Cotty P.J. (2019). “Ground-truthing” efficacy of biological control for aflatoxin mitigation in farmers’ fields in Nigeria: From field trials to commercial usage, a 10-Year study. Front. Microbiol..

[234] Senghor L.A., Ortega-Beltran A., Atehnkeng J., Callicott K., Cotty P., Bandyopadhyay R. (2019). The atoxigenic biocontrol product Aflasafe SN01 is a valuable tool to mitigate aflatoxin contamination of both maize and groundnut cultivated in Senegal. Plant Disease. https://apsjournals.apsnet.org/doi/pdf/10.1094/PDIS-03-19-0575-RE.

