# Supplementary material for: Aflatoxins: Producing-Molds, Structure, Health Issues and Incidence in Southeast Asian and Sub-Saharan African Countries
Source: Int J Environ Res Public Health. 2020 Feb 13;17(4):1215. doi: 10.3390/ijerph17041215 (PMC7068566; doi:10.3390/ijerph17041215)
Supplement: Supplementary file 1 [file ijerph-17-01215-s001.pdf]

International Journal of
Environmental Research and Public Health

MDPI

Review

Aflatoxins: Producing-Molds, Structure, Health Issues and Incidence in Southeast Asian and Sub-Saharan African Countries

Noreddine Benkerroum †

Table S1. Key properties of aflatoxins and their metabolites. Data compiled from PubChem of the National Center for Biotechnology Information [1] and ChemSpider of the Royal Society of Chemistry [2] databases, unless references are indicated beside the data.

Aflatoxin	MW (g/mol)	Formula	Melting Point (°C) [a]	Toxicity			Adverse Health Effects [b]
				LD$_{50}$ (mg/kg bw)	Test Organism	Route	
Aflatoxin B1	312.063	$C_{17}H_{12}O_6$	268.5	0.24–60 [3]<br>3.0	Various animals and chick embryo<br>Human	Oral, intraperitoneal or injection in chick embryo<br>In vitro experiments	Hepatotoxicity, genotoxicity, carcinogenicity, immuno-toxicity, teratogenicity
Aflatoxin B2	314.079	$C_{17}H_{14}O_6$	286–289 [3]	1.7	Duck	Oral	Week mutagenicity, hepatotoxicity, and carcinogenicity [4]
Aflatoxin B$_{2a}$	330.074	$C_{17}H_{14}O_7$	240 [3]	>400 µg showed a weak toxicity [5,6]	Ducklings	Oral	Low toxicity (200-fold less than B1) [6,7]
Aflatoxin M1	328.058	$C_{17}H_{12}O_7$	297–299	0.32<br>1.5	Duck<br>Rat	Unreported<br>Oral	Hepatotoxicity, nephrotoxicity, carcinogenicity
Aflatoxin M2	330.074	$C_{17}H_{14}O_7$	237–240	3.1 [8]	Ducklings [8]	Oral [8]	Same as M1 but to a lesser extent
Aflatoxin P1	298.048	$C_{16}H_{10}O_6$	240	>150 mg/kg<br>> 190 ng/egg [3]	Mouse<br>Chick embryo [3]	Intraperitoneal<br>Injection [3]	Same as B1 but to a lesser extent
Aflatoxin Q1	328.058	$C_{17}H_{12}O_7$	250	207 ng/egg [9]<br>NR	Chick embryo [9]<br>Bacteria [4]	Injection [9]<br>Ames' test [4]	Non-carcinogenic on fish [4]<br>50-fold less mutagenic than B1
Aflatoxicol (R$_0$) [c]	314.079	$C_{17}H_{14}O_6$	230–234 [3]	NA<br>NR	NA<br>Bacteria	NA<br>Ames test	Hepatotoxicity, carcinogenicity and mutagenicity. Forms the same DNA-adduct as B1. Two to 18-fold less toxic than B1 [4,10–15]
Aflatoxicol M1 [d]	330.074	$C_{17}H_{14}O_7$	215.31 (predicted)	NA<br>NR	NA<br>Bacteria [16]	NA<br>Ames' test [16]	Low toxicity, mutagenicity, and carcinogenicity [4,17]
Aflatoxicol H1 [d]	330.074	$C_{17}H_{14}O_7$	NA	Not toxic [18]<br>NR	Chick embryo [18]<br>Bacteria [18]	Injection 104]<br>Ames' test [18]	Weekly toxic to inactive<br>(A detoxified form of B1) [19]
Aflatoxin G1	328.058	$C_{17}H_{12}O_7$	244–246	0.8 [8]	Duckling	Oral	Hepatotoxicity, nephrotoxicity, Carcinogenicity (animals)

Aflatoxin G2	330.074	$C_{17}H_{14}O_7$	237–240<br>226–229	2.5 [8]<br>Weekly mutagenic	Duckling<br>*S. typhimurium*	Oral<br>Ames' test	Low toxicity, no evidence for carcinogenicity in animals [4,19,20]
Aflatoxin $G2_a$ [d]	346.069	$C_{17}H_{14}O_8$	243.13<br>(Predicted)	NA	NA	NA	Low toxicity to inactive (a detoxified form of G1) [4,19]
Aflatoxin GM1	344.053	$C_{17}H_{12}O_8$	276	NA	NA	NA	NA
Aflatoxin GM2	346.069	$C_{17}H_{14}O_8$	270–272	NA	NA	NA	NA
Parasiticol	302.079	$C_{16}H_{14}O_6$	233.4–234.1[17]	05.0 to 10.0 µg/egg<br>50.0 µg/duck [17]	Chick embryo<br>Duckling [17]	Injection<br>Oral [17]	Lower toxicity than G1<br>Same acute toxicity as B1. No or weak carcinogenicity [17]
Aspertoxin	354.074	$C_{19}H_{14}O_7$	NA	0.7 µg/egg [21]	Chick embryo [21]	Injection [21]	Teratogenic on chicken. Same fatality rate in chick embryo as B1[21]

[a] Data collected from ChemSpider website (http://www.chemspider.com) unless indicated by an imbedded citation; [b] In the latest classification of mycotoxins, the IARC stated that there is "sufficient evidence" for the carcinogenicity of aflatoxins B1, G1, and M1 in experimental animals, but there is "limited evidence" or "insufficient evidence" in experimental animals for the carcinogenicity of aflatoxins B2 and G2, respectively; however, in view of mechanistic studies showing the ability of the major aflatoxins (B1, G1, B2, G2, M1) to form DNA adducts as a first step in genotoxicity, they were classified in group 1 carcinogens [20]; [c] Usually designated as the aflatoxin B1 reservoir, as it readily converts back to B1 by action of a dehydrogenase; [d] Mutagenicity induced in *Salmonella typhimurium* is <1% that of aflatoxin B1 taken as a reference [4]. *Abbreviations:* NA: Not available; NR: Not relevant; bw: Body weight.

Table S2. Incidence (%) and concentrations (μg/kg) of aflatoxins in staple agricultural products of selected countries from Sub-Saharan Africa. Data are for total aflatoxins (B1+B2+G1+G2), unless otherwise stated in the footnotes.

Country	AEZ	Peanut/Groundnut		Maize		Millet		Sorghum		Sunflower		References
	(Climate type) [a]	Mean [b] (Range)	+ve (%)	Mean (Range)	+ve (%)	Mean (Range)	+ve (%)	Mean (Range)	+ve (%)	Mean (Range)	+ve (%)	
Uganda	Kioga planes [c] (Am)	7.3–221 (2.5–450)	20–60	25.4–71 (4.5–180)	50–80	-	-	61–170 (4–26)	80–100	-	-	[22]
	Western (Aw)	7.0 (3–13)	25	75.2 (3.5–248)	95	-	-	-	-	-	-	
	Savannah (Aw)	8.8 (5.5–12)	30	26.56 (3.3–105)	100	-	-	11.5 (29–472)	100	-	-	
	Grasslands (Aw)	85.4 (2.5–175)	30	46.0 (3.1–510)	95	-	-	102.3 (28–227)	100	-	-	
	South-East (Af)	-	-	-	-	14.0 (NS-NS)	100	-	-	-	-	[23]
	Commercial [d]	181 (0–849)	82	-	-	-	-	-	-	-	-	[24]
Kenya	Tharaka-Nithi (Cbw)	-	-	24[e(R)] (<1–537)	88[e(R)]	11[e(R)] (<1.0–152)	64[e(R)]	1.2[e(R)] (<1–18)	33[e(R)]	-	-	[25]
		-	-	23.9[e(D)] (<1.0–775)	75.4[e(D)]	66[e(D)] (<1–1658)	69[e(D)]	1.5[e(D)] (<1.0 – 23.1)	85[e(D)]	-	-	
	Kisii (Af and Cfb)	NS (1.6–591)	100	-	-	-	-	-	-	-	-	[26]
		-	-	4.0[e(R)] (<1–103)	46.8[e(R)]	0.1[e(R)] (<1–3.0)	21[e(R)]	0.9[e(R)] (<1–16.4)	11[e(R)]	-	-	[25]
		-	-	8.9[e(D)] (<1.0–372)	78[e(D)]	0.5[e(D)] (<1–2.9)	77[e(D)]	-	-	-	-	
		-	-	28.5[e] (<1.0–559)	76.3	-	-	-	-	-	-	[27]
	Migori (Am)	-	-	12.7[e] (0.98–121)	56	-	-	-	-	-	-	
	Bungoma (Cfb)	-	-	7.9[e(R)] (<1–218)	81[e(R)]	0.6[e(R)] (0–2.9)	98[e(R)]	3.5[e(R)] (<1–92)	97[e(R)]	-	-	[25]
		-		3.5[e(D)] (<1.0–39.3)	72[e(D)]	0.9[e(D)] (<1.0 – 13.8)	75[e(D)]	0.9[e(D)] (<1.0–12.3)	84[e(D)]			

Isiolo<br>(Aw)	-	-	9.6[e(R)]<br>(<1–121)	98[e(R)]	-	-	3.8[e(R)]<br>(<1–12.8)	100[e(R)]	-	-	
	-	-	67.3[e(D)]<br>(<1.0–1137)	50[e(D)]	-	-	2.0[e(D)]<br>(<1.0–11.9)	57[e(D)]	-	-	
Kwale<br>(As)	-	-	29[e(R)]<br>(<1–394)	97[e(R)]	-	-	-	-	-	-	
	-	-	3.5[e(D)]<br>(<1.0 – 19.2)	95[e(D)]	-	-	-	-	-	-	
Eldoret<br>(Cfb)	1147<br>(NS-NS)	NS	-	-	-	-	-	-	1524<br>(NS-NS)	NS	[28]
Nandi<br>(Aw)			1.3[f]<br>(0–3.92)	-	-	-	-	-	-	-	[29]
	-	-	0.98<br>(0.1–5.3)	68	1.6<br>(0.14–11)	92	24.5<br>(0.15–210)	66	-	-	[30]
Makueni<br>(Aw)	-	-	24.8<br>(0.1–279)	80	17.2<br>(0.4–231)	82	17.8<br>(0–265)	86	-	-	
Busia<br>(Am)	NS<br>(0.1–268)	97.1	-	-	-	-	-	-	-	-	[26]
	NS<br>(>20–7525)	7.5	-	-	-	-	-	-	-	-	[31]
Homabay<br>(Aw)	-	-	24.5[e]<br>(0.98–722)	56	-	-	-	-	-	-	[27]
Kitale<br>(Cfb)	-	-	9.7<br>(0–72)	70	-	-	-	-	-	-	[32]
Nakuru<br>(Cfb)	-	-	4.2<br>(0–13)	97	-	-	-	-	-	-	
Makueni-Kitui<br>(Aw, BSh)	-	-	9. 1[g(Gm)]<br>(0–48,000)	35[h]	-	-	-	-	-	-	[33]
Korogocho<br>(Cwb)	-	-	6.7<br>(0–89)	NS	-	-	8.1<br>(0.2–194)	NS	-	-	[34]
Dagoretti<br>(Cfb)	-	-	3.0<br>(0–20)	NS	-	-	2.6<br>(0.1–15)	NS	-	-	
Makueni<br>(BSh, Aw, Cwb)	-	-	52.9[g(Gm)]<br>(<1–5400)	59[h]	-	-	-	-	-	-	[35]
	-	-	39[e]<br>(0.01–1455)	68	-	-	-	-	-	-	[27]

		-	-	24.8 (0.05–279)	80	17.2 (0.4–231)	82	17.8 (0.04–265)	86	-	-	[36]
	Embu (Aw)	-	-	196.3[e] (0.95–9092)	64	-	-	-	-	-	-	[27]
	Kitui (BSh and Aw)	-	-	0.7 (0–13)	97	-	-	-	-	-	-	[32]
		-	-	35.3[g(Gm)] (<1–25,000)	45[h]	-	-	-	-			[35]
	Machakos (Cwb)	-	-	17.8[g(Gm)] (<1–3800)	52[h]	-	-	-	-	-	-	
		-	-	11[e] (1.3–71)	61	-	-	-	-	-	-	[27]
	Thika (Cwb)	-	-	7.52[g(Gm)] (<1.0–46,400)	25[h]	-	-	-	-	-	-	[35]
	Commercial[4]	NS (>4.0–32,328)	49	-	-	-	-	-	-	-	-	[37]
Tanzania	Long, Babati (Cwb)	-	-	2.6 (2.1–3.6)	17	-	-	-	-	-	-	[38]
	Sabilo, Babati (Cwb)	-	-	3.32 (2.2–26)	28	-	-	-	-	-	-	
	Seloto, Babati (Cwb)	-	-	2.62 (2.1–4.0)	13	-	-	-	-	-	-	
	Tabora (Aw)	-	-	NS (5–158)	37	-	-	-	-	-	-	[39]
	Kilimanjaro (Cwb)	-	-	NS (1.0–80)	20	-	-	-	-	-	-	
	Ruvuma (Aw)	-	-	NS (7–26)	6	-	-	-	-	-	-	
	Iringa (Cwb)	-	-	NS (13–58)	7	-	-	-	-	-	-	
	Kilosa (Aw)	-	-	106 (3.0–1081)	18	-	-	-	-	-	-	[40]
	Hanang' (Csb)	-	-	4.0 (3.0–5.0)	8	-	-	-	-	-	-	
	Rungwe (Cwb)	-	-	5 (2–8)	4	-	-	-	-	-	-	
	Chitego	21.9	NS	-	-	-	-	9.1	NS	19.0	NS	[41]

	(Aw)	(0–56)						(0–62.5)		(0–605)		
	Laikala (Bsh)	84.9 (0–427)	NS	0.76 (0–1.2)	NS	-	-	2.7 (0–29.8)	NS	61.1 (0–489)	NS	
	Mlali (Bsh)	85.4[e] (0–298)	NS	2.8[e] (0–22)	NS	-	-	25.7[e] (0–70)	NS	4.9[e] (0–44)	NS	
	Moleti (Bsh)	377.3[e] (0–3297)	NS	4.2[e] (0–43)	NS	-	-	9.4[e] (0–73.9)	NS	100[e] (0–425)	NS	
	Njoro (Csb)	289.7[e] (0–1179)	NS	2.5[e] (0–29.2)	NS	-	-	93.3[e] (0–138.7)	NS	82.0[e] (0–295)	NS	
	Babati (Cwb)	-	-	-	-	-	-	-	-	46.8[i] (1.8–162)	83	
	Singida (BSh)	-	-	-	-	-	-	-	-	45.8[i] (1.4–262)	83	
	Dodoma (BSh)	-	-	-	-	-	-	-	-	59.6[i] (1.7–281)	71	[42]
	Mbeya (Cfa)	-	-	-	-	-	-	-	-	21[i] (1.4–174)	89	
	Morogoro (Aw)	-	-	-	-	-	-	-	-	119[i] (2.8–663)	50	
Zambia	Chipata (Aw)	451 (>1–4000)	NS	-	-	-	-	-	-	-	-	[43]
	Petauke (Cwa)	4.34 (>1–10)	NS	-	-	-	-	-	-	-	-	
	Ndola (Cwa)	242 (>1–1600)	NS	-	-	-	-	-	-	-	-	
	Katete (Cwa)	13.6 (>1–74)	NS	-	-	-	-	-	-	-	-	
	Kitwe (Cwa)	499 (>1–11,100)	NS	-	-	-	-	-	-	-	-	

	Kabwe (Cwa)	21.4 (>1–145)	NS	-	-	-	-	-	-	-	-	
	Southern area[j] (BSh, Cwa)	22.0 (3.9–621)[k]	100[l]	12.0 (3.9–621)[k]	73[l]	-	-	-	-	-	-	[44]
	Central area[m] (Cwa)	90 (0–3420)[k]	51[l]	11.0 (0–3420)[k]	42[l]	-	-	-	-	-	-	[44]
South Africa	Northen area[n] (Cwb)	6.0 (0–1416)[k]	27[l]	25 (0–1416)[k]	22[l]	-	-	-	-	-	-	[45]
South Africa	Limpopo (BSh)	-	-	48[e] (0–133)	20	-	-	-	-	-	-	[45]
South Africa	Mpumalanga (Cfb)	-	-	1 (1–2)[e]	6.5	-	-	-	-	-	-	[45]
South Africa	Commercial[d]	14 (0–74)	90	-	-	-	-	-	-	-	-	[46]
South Africa	All regions	ND	0	-	-	-	-	-	-	-	-	[47]
Nigeria	Kano (BSh)	6.0[o(LB)] (<0.1–97)	26	-	-	-	-	-	-	-	-	[48]
Nigeria	Lagos (Aw)	-	-	0.6[o(LB)] (0–5)	10	-	-	-	-	-	-	[48]
Nigeria	Sokoto (BSh)	96.0 (1–415)	-	-	-	-	-	-	-	-	-	[49]
Nigeria	Isa (BSh)	64.0 (2.0–317)	-	-	-	-	-	-	-	-	-	[49]
Nigeria	Tambuwal (BSh)	92.9 (0.9–646)	-	-	-	-	-	-	-	-	-	[49]
Nigeria	Ogun (Aw)	-	-	300 (NS-NS)	NS	34.3 (NS-NS)	NS	221 (NS-NS)	NS	-	-	[50]
Nigeria	Lagos (Aw)	-	-	603 (NS-NS)	NS	120.5 (NS-NS)	NS	1245 (NS-NS)	NS	-	-	[50]
Nigeria	South-East (Am, Aw)	-	-	43[(Gm)] (2.7–1460)	87.5[p]	-	-	-	-	-	-	[51]
Nigeria	Western states (Aw)	-	-	200 (25–770)	45	-	-	-	-	-	-	[52]
Nigeria	Suleja and Tafa (Aw)	-	-	-	-	-	-	225[e(ML)] (0–728)	64	-	-	[53]
Nigeria	Borgu and Magama (Aw)	-	-	-	-	-	-	210[e(ML)] (0–712)	55	-	-	[53]

	Minna Mokowa (Aw)	-	-	-	-	-	-	165[e(ML)] (0–721)	57	-	-	
	Mariga-Rafi-Wushishi (Aw)	-	-	-	-	-	-	198[e(ML)] (0–1164)	45	-	-	
Cameroun	South-West (Am, Af)	26[e] (6.0–125)	NS	100[e] (6–645)	NS	-	-	-	-	-	-	[54]
	South-East (Am)	22[e] (6.0–77)	NS	96[e] (6–216)	NS	-	-	-	-	-	-	
	Western highland (Aw)	22[e] (6.0–110)	NS	47[e] (6–210)	NS	-	-	-	-	-	-	
Ghana	Ashanti (Aw, HF)	2.2 (0–17)	NS	6 (0–135)	NS	-	-	-	-	-	-	[55]
	Brong Ahafo (Aw, HF)	5.5 (0–54)	NS	0.6 (0–9)	NS	-	-	-	-	-	-	
	Volta (Aw, HF)	42.4 (0–387)	NS	9.0 (0–83)	NS	-	-	-	-	-	-	
	Brong Ahafo (Aw, DS)	145.6 (0–1999)	NS	16.8 (0–226)	NS	-	-	-	-	-	-	
	Northern (Aw, DS)	78 (0–3868)	NS	15.9 (0–341)	NS	-	-	-	-	-	-	
	Upper East (Aw, SGS)	0.3 (0–1.0)	NS	15.4 (0–82)	NS	-	-	-	-	-	-	[56]
	Upper West (Aw, SGS)	15.9 (0–181)	NS	16.4 (0–190)	NS	-	-	-	-	-	-	
	Akomadan (Aw, FRT)	-	-	NS (0–112)	83	-	-	-	-	-	-	[57]
	Ejura (Aw, FRT)	-	-	NS (1–945)	100	-	-	-	-	-	-	
	Wenchi (Aw, SVT)	-	-	NS (0–23)	71	-	-	-	-	-	-	
	Fumesua (Aw, RFR)	-	-	NS (0–692)	78	-	-	-	-	-	-	
	Commercial[d]	-	-	38.7 (3–275)	42	-	-	14 (6–19)	25	-	-	[56]
Togo	Volta (Aw, DS)	0.3 (0–1.0)	NS	24.2 (0–157)	NS	-	-	-	-	-	-	[56]
	Northern (Aw, SGS)	34.9 (0–168)	NS	6.8 (0–59)	NS	-	-	-	-	-	-	

Benin	Littoral (Aw)	$7.6^{o(LB)}$ (<0.1–105)	19	-	-	-	-	-	-	-	-	[48]
	Borgou (Aw)	-	-	$1.6^{o(LB)}$ (<0.1–20)	32	-	-	-	-	-	-	
Mali	Bamako (Aw)	$9.4^{o(LB)}$ (<0.1–246)	15	-	-	-	-	-	-	-	-	
	Sikasso (Aw)	$2.2^{o(LB)}$ (<0.1–43)	29	-	-	-	-	-	-	-	-	
Mozambique	Amendo	362.2 (0–2740)	NS									[58]
	Mugovola (Aw)	230.7 (0–1382)	NS									
	Murrupuia (Aw)	750.8 (0–1320)	NS									
	Erati (Aw)	20.5 (0–167)	NS									[59]
	Manhica-Magude (Aw)	0.82 (NS-NS)	4.3									
	Massinga (Aw)	1.35 (NS-NS)	2.4									
	Morrumbene (Aw)	3.13 (NS-NS)	7.2									
	Inharrime (Aw)	3.50 (NS-NS)	5.2									
	Homoine-Maxixe (Aw)	5.12 (NS-NS)	13.4									
	Zavala (Aw)	6.62 (NS-NS)	11.9									

[a] The type of climate (in the parenthesis) is defined according to Köppen-Geiger classification [60]: Cfb: Warm temperate (C) fully humid (f) warm summer (b); Cwa: Warm temperate (C) winter dry (w) hot summer (a); Cwb: Warm temperate (C) winter dry (w) warm summer (b); Af: Tropical (A) fully humid (f); Aw: Tropical (A) winter dry (w); As: Tropical (A) steppe (s); Am: Tropical (A) monsoonal (m), Csb: Warm temperate (C) steppe (s) warm summer (b); BSh: Arid (B) steppe (S) hot (h), Cfa: Warm temperate (C) fully humid (f) hot summer (a), [b] Arithmetic mean as a default, geometric mean or median when followed by Gm or Md, respectively; [c] Different regions each has its own mean, minimum and maximum, and incidence values, [d] Commercial samples can be from different origins and, hence, their aflatoxin contents may reflect their origin and the storage conditions rather than the area where they are sold, [e] Data are relative to the occurrence of aflatoxin B1; either in the [(R)]rainy season or the [(D)]dry season; [f] Results field training for farmers with supervised application of the good agricultural practices; [g] Exceptionally high aflatoxin levels recorded in 2004 during a major aflatoxicosis in Kenia; [h] Percentage for samples containing more than 20 mg/kg of aflatoxins; [I] Samples collected from micro- and small-scale sunflower oil processors during the harvesting season of 2014; [j] Rainfall below 800 mm, high temperature (30 °C); [k] The highest and lowest aflatoxin concentrations were not discriminated between peanut and maize samples by the authors; [l] Percentages were calculated for samples containing more than 4.0 μg/kg of aflatoxins; [m] High rainfall (900–1300 mm),

moderate temperature (23–25 °C); [n] High rainfall, cool temperature (16 °C); Total aflatoxins (AFB1+AFB2+AFG1 = AFG2); [p] Percentage of samples contaminated with levels exceeding with more than 4 µg/kg after 4 months of storage. *Abbreviations and symbols*: AEZ: Argo-ecological zone; +ve; Positive samples (aflatoxin levels higher than the level of detection LOD unless specified otherwise); "-" no available data, 0 "zero": aflatoxin level below LOD; HF = Humid Forest, DS = Derived Savanna, and SGS = Southern Guinea Savanna. SVT = Savana Transition; RFR = Rain Forest; FRT = Forest Transition; ML: Mouldy samples (biased sampling procedure was used by the authors); LB: Lower bound (scenario where the concentration of non-detected analyte is zero and the concentration of detected but non-quantified analyte is the limit of detection)

Table S3. Incidence and concentrations (µg/kg) of aflatoxin contamination of staple crops in selected countries from the Southeast Asian region. Data are for total aflatoxins (B1+B2+G1+G2), unless otherwise stated in the footnotes.

Country	AEZ (Climate Type) [a]	Peanut/Groundnut		Maize		Rice		Sorghum		References
		Mean [b] (Range)	+ve (%)	Mean [b] (Range)	+ve (%)	Mean (Range)	+ve (%)	Mean (Range)	+ve (%)	
India	20 states (Various)	-	-	-	-	NS (0.1–308) [c]	68	-	-	[61]
	Karnataka (BSh, Aw)	510.7 [c] (NS-NS)	NS	67.3 [c] (201–714)	100	-	-	882 [c] (582–1250)	100	[62]
	Eastern region (Cwa, Aw)	-	-	<5 [c(Md)] (0–120)	47 [d]	-	-	-	-	[63]
	Western region (BSh)	-	-	15 [c(Mm)] (0–333)	53 [d]	-	-	-	-	
	North (BSh, Cwa)	-	-	30 [c(Md)] (0–666)	69 [d]	-	-	-	-	
	Southern region (Aw)	-	-	<5 [c(Md)] (0–400)	21 [d]	-	-	-	-	
	Mahashtra (BSh, Aw, Am)	-	-	-	-	-	-	NS (0.49–139)	82	[64]
	Rajasthan (BWh, BSh)	-	-	-	-	-	-	NS (0.1–15)	86	
	Tamil Nadu (Aw)	-	-	-	-	-	-	NS (0.01–264)	88	
	Punjab (Af)	-	-	-	-	NS (0->30.0)	91	-	-	[65]
Nepal	Eastern region	NS	34	NS	32	-	-	-	-	[66]

	(Cfa)	(54–1806)		(64–859)						
The Philippines	NS	58 (0–885)	65	76 (0.0–1152)	95	-	-	-	-	[67]
	Iloco (Aw)	-	-	22 (NS–30)	NS	-	-	-	-	[68]
		-	-	39 (NS–1215)	NS	-	-	-	-	
	South Catabato (Af, Aw)	-	-	68.0 (NS–178)	NS	-	-	-	-	
	Commercial	-	-	-	-	1.5 (0–8.7)	95	-	-	[69]
Thailand	Northeast (Aw)	-	-	-	-	0.8 0–13.4	63	-	-	[70]
	Central region (Aw)	-	-	-	-	1.7 (0–26.6)	53	-	-	
	Singburi (Aw)	245 $^{(R)}$ (NS-NS)	56	-	-	-	-	-	-	[71]
		139 $^{(D)}$ (NS-NS)	41	-	-	-	-	-	-	
		28 $^{(H)}$ (NS-NS)	91	-	-	-	-	-	-	
	Ratburi (Aw)	329 $^{(R)}$ (NS-NS)	63	-	-	-	-	-	-	
		71 $^{(D)}$ (NS-NS)	63	-	-	-	-	-	-	
		99 $^{(H)}$ (NS-NS)	72	-	-	-	-	-	-	
	Songkhla (Am)	207 $^{(R)}$ (NS-NS)	47	-	-	-	-	-	-	
		96 $^{(D)}$ (NS-NS)	70	-	-	-	-	-	-	
		62 $^{(H)}$ (NS-NS)	68	-	-	-	-	-	-	
	Whole country	1563 $^{(R)}$	NS	-	-	-	-	-	-	[72]

		(0–12,256)								
		1811$^{(D)}$ (0–9500)	NS	-	-	-	-	-	-	
		1203$^{(H)}$ (0–7660)	NS	-	-	-	-	-	-	
		1530 (0–12,256)	49	400 (0–2730)	39	67 (0–248)	2	-	-	
	Commercial	47 (0–304)	80	196 (0–750)	NS	-	-	-	-	[73]
		31.5 (2.2–171)	NS	-	-	-	-	-	-	[74]
	Penang Island (Af)	NS (17–711)	43	-	-	-	-	-	-	[75]
		-	-	-	-	NS (1.1–5.2)	NS	-	-	[76]
	NS	NS (20–1000)	16	-	-	-	-	-	-	[77]
Malaysia	Commercial	11.3 (0–103)	79	-	-	-	-	-	-	[78]
	Commercial	-	-	-	-	NS (0.15–4.4)	25	-	-	[79]
	Commercial	4.3^{c} (1.5–15.3)	85	-	-	1.75^{c} (0.7–3.8)	70	-	-	[80]
	East Java (Aw)	-	-	149 (NS-390)	100	-	-	-	-	
Indonesia	Lampung (Af)	-	-	144 (0–350)	92	-	-	-	-	[77]
	Commercial	-	-	464 (NS-490)	100	-	-	-	-	
	Hanoi (Cwa)	9.28 (>0.1–16.0)	25	2.62 (>0.1–25.0)	35	0.42 (>0.1–13)	10			
Vietnam	Thanh Hoa (Cwa, Aw)	4.96 (>0.1–159)	26	5.39 (>0.1–94.5)	26.5	2.04 (>0.1–93)	12.5			[81]
	Ha Giang (Cwa)	16.57 (>0.1–362)	20.7	66.1 (>0.1–1572)	30	1.01 (>0.1–26)	5.4			

Captions and abbreviations are as defined in the footnotes of Table S2, unless otherwise specified herein ; [a] The type of climate (in the parenthesis) is defined according to Köppen-Geiger [60]. [b] Arithmetic mean as a default value or geometric mean[(Gm)] or median[(Md)]; [c] Percentage of samples containing more than 5.0 µg/kg of aflatoxin; [(R)] Rainy season; [(H)] Hot season; [(D)] Dry season.

References

1. Pubchem. Explore chemistry. 2020. Accessed: 11 February 2020; Available from: https://pubchem.ncbi.nlm.nih.gov.
2. Chemspider. Search and share chemistry. 2020. Accessed: 11 February 2020; Available from: http://www.chemspider.com.
3. Lai, D.Y.; Woo, Y.T.; Arcos, J.C.; Argus, M.F. Difuroxanthone-, difurocoumarolactone- and difuroanthraquinone-type alkylating agents: Carcinogenicity and structure activity relationships : Other biological properties : Metabolism : Environmental significance. 1985. Accessed: 16 August, 2019. https://www.epa.gov/nscep
4. Wong, J.J.; Hsieh, D.P. Mutagenicity of aflatoxins related to their metabolism and carcinogenic potential. *Proc. Natl. Acad. Sci. U. S. A.* **1976**, *73*, 2241–2244.
5. Dutton, M.F.; Heathcote, J.G. The structure, biochemical properties and origin of the aflatoxins B2a and G2a. *Chem. Ind.* **1968**, *13*, 418–21.
6. Lillehoj, E.B.; Ciegler, A. Biological activity of aflatoxin B2a. *Appl. Microbiol.* **1969**, *17*, 516–519.
7. Dutton, M.F. Enzymes and aflatoxin biosynthesis. *Microbiol. Rev.* **1988**, *52*, 274–295.
8. Applebaum, R.S.; Brackett, R.E.; Wiseman, D.W.; Marth, E.H. Aflatoxin: Toxicity to dairy cattle and occurrence in milk and milk products - A review. *J. Food Prot.* **1982**, *45*, 752–777.
9. Hsieh, D.P.H.; Salhab, A.S.; Wong, J.J.; Yang, S.L. Toxicity of aflatoxin Q1 as evaluated with the chicken embryo and bacterial auxotrophs. *Toxicol. Appl. Pharmacol.* **1974**, *30*, 237–242.
10. Karabulut, S.; Paytakov, G.; Leszczynski, J. Reduction of aflatoxin B1 to aflatoxicol: A comprehensive DFT study provides clues to its toxicity. *J. Sci. Food Agric.* **2014**, *94*, 3134–3140.
11. Hendricks, J.D.; Nixon, J.E.; Pawloswki, N.E.; Loveland, P.M.; Sinnhuber, R.O. Carcinogenicity of Aflatoxlcol in Fischer 344 Rats2, 3, 4. *JNCI: Journal of the National Cancer Institute* **1981**, *66*, 1159–1163.
12. Frazzoli, C.; Gherardi, P.; Saxena, N.; Belluzzi, G.; Mantovani, A. The hotspot for (Global) one health in primary food production: Aflatoxin M1 in dairy products. *Front Public Health* **2017**, *4*.
13. Verheecke, C.; Liboz, T.; Mathieu, F. Microbial degradation of aflatoxin B1: Current status and future advances. *Int. J. Food Microbiol.* **2016**, *237*, 1–9.
14. Theumer, M.G.; Henneb, Y.; Khoury, L.; Snini, S.P.; Tadrist, S.; Canlet, C.; Puel, O.; Oswald, I.P.; Audebert, M. Genotoxicity of aflatoxins and their precursors in human cells. *Toxicol. Lett.* **2018**, *287*, 100–107.
15. Salhab, A.S.; Edwards, G.S. Comparative in vitro metabolism of aflatoxicol by liver preparations from animals and humans. *Cancer Res.* **1977**, *37*, 1016–21.

16. Loveland, P.M.; Coulombe, R.A.; Libbey, L.M.; Pawlowski, N.E.; Sinnhuber, R.O.; Nixon, J.E.; Bailey, G.S. Identification and mutagenicity of aflatoxicol-M1 produced by metabolism of aflatoxin B1 and aflatoxicol by liver fractions from rainbow trout (Salmo gairdneri) fed beta-naphthoflavone. *Food Chem Toxicol* **1983**, *21*, 557–62.
17. Stubblefield, R.D.; Shotwell, O.L.; Shannon, G.M.; Weisleder, D.; Rohwedder, W.K. Parasiticol: a new metabolite from *Aspergillus parasiticus*. *Agr Food Chem* **1970**, *18*, 391–393.
18. Salhab, A.S.; Hsieh, D.P. Aflatoxicol H1: a major metabolite of aflatoxin B1 produced by human and rhesus monkey livers in vitro. *Res. Commun. Chem. Pathol. Pharmacol.* **1975**, *10*, 419–429.
19. Deshpande, S. Fungal toxins. In *Handbook of Food Toxicology*; CRC Press: Boca Raton: CRC Press, 2002, pp. 387–456.
20. IARC (International Agency for Research on Cancer). Chemical agents and related occupations. Aflatoxins. In *IARC Monographs on the Evaluation of Carcinogenic Risks to Humans*; World Health Organisation: Lyon (Fr), 2012; Volume 100F, pp. 225–248.
21. Rodricks, J.V.; Henery-Logan, K.R.; Campbell, A.D.; Stoloff, L.; Verrett, M.J. Isolation of a New Toxin from Cultures of Aspergillus flavus. *Nature* **1968**, *217*, 668–668.
22. Lukwago, F.B.; Mukisa, I.M.; Atukwase, A.; Kaaya, A.N.; Tumwebaze, S. Mycotoxins contamination in foods consumed in Uganda: A 12-year review (2006–18). *Scientific African* **2019**, *3*, e00054.
23. Kitya, D.; Bbosa, G.S.; Mulogo, E. Aflatoxin levels in common foods of South Western Uganda: a risk factor to hepatocellular carcinoma. *European Journal of Cancer Care* **2010**, *19*, 516–521.
24. Baluka, S.A.; Schrunk, D.; Imerman, P.; Kateregga, J.N.; Camana, E.; Wang, C.; Rumbeiha, W.K. Mycotoxin and metallic element concentrations in peanut products sold in Ugandan markets. *Cogent Food & Agriculture* **2017**, *3*, 1313925.
25. Sirma, A.J.; Senerwa, D.M.; Grace, D.; Makita, K.; Mtimet, N.; Kang'ethe, E.K.; Lindahl, J.F. Aflatoxin B1 occurrence in millet, sorghum and maize from four agro-ecological zones in Kenya. *Afr J Food Agric Nutr Dev* **2016**, *16*, 10991–11003.
26. Menza, N.; Muturi, M.; Kamau M, L. Incidence, types and levels of aflatoxin in different peanuts varietiesproduced in Busia and Kisii central districts, Kenya. *Open J Med Microbiol* **2015**, *5*, 209–221.
27. Mahuku, G.; Nzioki, H.S.; Mutegi, C.; Kanampiu, F.; Narrod, C.; Makumbi, D. Pre-harvest management is a critical practice for minimizing aflatoxin contamination of maize. *Food Control* **2019**, *96*, 219–226.
28. Nyandieka, H.S.; Nyamogoba, H.D.; Nyamwange, C.I. Distribution of aflatoxins and micro organisms in peanut and sunflower seed products and their potential health hazards. *Pak. J. Med. Res.* **2014**, *53*, 67–70.
29. Marete, N.G.; Kanja, W.L.; Mbaria, M.J.; Okumu, O.M.; Ateku, A.P.; Korhonen, H.; Joutsjoki, V. Effects of the Use of good agricultural practices on aflatoxin levels in maize grown in Nandi county, Kenya. *Sci* **2019**, *1*.

30. Kang'ethe, E.K.; Sirma, A.J.; Murithi, G.; Mburugu-Mosoti, C.K.; Ouko, E.O.; Korhonen, H.J.; Nduhiu, G.J.; Mungatu, J.K.; Joutsjoki, V.; Lindfors, E.; et al. Occurrence of mycotoxins in food, feed, and milk in two counties from different agro-ecological zones and with historical outbreak of aflatoxins and fumonisins poisonings in Kenya. *Food Qual Saf* **2017**, *1*, 161–170.
31. Mutegi, C.K.; Cotty, P.J.; Bandyopadhyay, R. Prevalence and mitigation of aflatoxins in Kenya (1960-to date). *World Mycotoxin J.* **2018**, *11*, 341–357.
32. Gachara, G.W.; Nyamache, A.K.; Harvey, J.; Gnonlonfin, G.J.B.; Wainaina, J. Genetic diversity of *Aspergillus flavus* and occurrence of aflatoxin contamination in stored maize across three agro-ecological zones in Kenya. *Agriculture & Food Security* **2018**, *7*, 52.
33. Daniel, J.H.; Lewis, L.W.; Redwood, Y.A.; Kieszak, S.; Breiman, R.F.; Flanders, W.D.; Bell, C.; Mwihia, J.; Ogana, G.; Likimani, S.; et al. Comprehensive assessment of maize aflatoxin levels in Eastern Kenya, 2005–2007. *Environ. Health Perspect.* **2011**, *119*, 1794–9.
34. Kiarie, G.; Dominguez-Salas, P.; Kang'Ethe, S.; Grace, D.; Lindahl, J. Aflatoxin exposure among young children in urban low-income areas of Nairobi and association with child growth. *Afr J Food Agr Nutr Develop* **2016**, *16*, 10967–10990.
35. Lewis, L.; Onsongo, M.; Njapau, H.; Schurz-Rogers, H.; Luber, G.; Kieszak, S.; Nyamongo, J.; Backer, L.; Dahiye, A.M.; Misore, A.; et al. Aflatoxin contamination of commercial maize products during an outbreak of acute aflatoxicosis in eastern and central Kenya. *Environ. Health Perspect.* **2005**, *113*, 1763–1767.
36. Kang'ethe, E.; Gatwiri, M.; Sirma, A.; Ouko, E.; Mburugu-Musoti, C.; Kitala, P.; Nduhiu, G.; Nderitu, J.; Mungatu, J.; Hietaniemi, V. Exposure of Kenyan population to aflatoxins in foods with special reference to Nandi and Makueni counties. *Food Qual Saf* **2017**, *1*, 131–137.
37. Mutegi, C.; Wagacha, M.; Kimani, J.; Otieno, G.; Wanyama, R.; Hell, K.; Christie, M.E. Incidence of aflatoxin in peanuts (*Arachis hypogaea* Linnaeus) from markets in Western, Nyanza and Nairobi Provinces of Kenya and related market traits. *J. Stor. Prod. Res.* **2013**, *52*, 118–127.
38. Nyangi, C.; Beed, F.; Mugula, J.; Boni, S.; Koyano, E.; Mahuku, G.; Sulyok, M.; Bekunda, M. Assessment of pre-harvest aflatoxin and fumonisin contamination of maize in Babati District, Tanzania. *African Journal of Food Agriculture Nutrition and Development* **2016**, *16*, 11039–11053.
39. Kimanya, M.E.; De Meulenaer, B.; Tiisekwa, B.; Ndomondo-Sigonda, M.; Devlieghere, F.; Van Camp, J.; Kolsteren, P. Co-occurrence of fumonisins with aflatoxins in home-stored maize for human consumption in rural villages of Tanzania. *Food Addit Contam: Part A* **2008**, *25*, 1353–1364.
40. Kamala, A.; Ortiz, J.; Kimanya, M.; Haesaert, G.; Donoso, S.; Tiisekwa, B.; De Meulenaer, B. Multiple mycotoxin co-occurrence in maize grown in three agro-ecological zones of Tanzania. *Food Control* **2015**, *54*, 208–215.
41. Seetha, A.; Munthali, W.; Msere, H.W.; Swai, E.; Muzanila, Y.; Sichone, E.; Tsusaka, T.W.; Rathore, A.; Okori, P. Occurrence of aflatoxins and its management in diverse cropping systems of central Tanzania. *Mycot. Res.* **2017**, *33*, 323–331.
42. Mmongoyo, J.A.; Wu, F.; Linz, J.E.; Nair, M.G.; Mugula, J.K.; Tempelman, R.J.; Strasburg, G.M. Aflatoxin levels in sunflower seeds and cakes collected from micro- and small-scale sunflower oil processors in Tanzania. *PLoS One* **2017**, *12*, e0175801.

43. Njoroge, S.M.C.; Matumba, L.; Kanenga, K.; Siambi, M.; Waliyar, F.; Maruwo, J.; Machinjiri, N.; Monyo, E.S. Aflatoxin B1 levels in groundnut products from local markets in Zambia. *Mycotoxin research* **2017**, *33*, 113–119.
44. Kachapulula, P.W.; Akello, J.; Bandyopadhyay, R.; Cotty, P.J. Aflatoxin contamination of groundnut and maize in Zambia: observed and potential concentrations. *J. Appl. Microbiol.* **2017**, *122*, 1471–1482.
45. Mngqawa, P.; Shephard, G.S.; Green, I.R.; Ngobeni, S.H.; de Rijk, T.C.; Katerere, D.R. Mycotoxin contamination of home-grown maize in rural northern South Africa (Limpopo and Mpumalanga Provinces). *Food Addit Contam: Part B* **2016**, *9*, 38–45.
46. Kamika, I.; Mngqawa, P.; Rheeder, J.P.; Teffo, S.L.; Katerere, D.R. Mycological and aflatoxin contamination of peanuts sold at markets in Kinshasa, Democratic Republic of Congo, and Pretoria, South Africa. *Food Addit Contam: Part B* **2014**, *7*, 120–126.
47. Meyer, H.; Skhosana, D.Z.; Motlanthe, M.; Louw, W.; Rohwer, E. Long term monitoring (2014–2018) of multi-mycotoxins in South African commercial maize and wheat with a locally developed and validated LC-MS/MS method. *Toxins (Basel)* **2019**, *11*.
48. Ingenbleek, L.; Sulyok, M.; Adegboye, A.; Hossou, E.S.; Koné, Z.A.; Oyedele, D.A.; K. J. Kisito, S.C.; Koreissi Dembélé, Y.; Eyangoh, S.; Verger, P.; et al. Regional Sub-Saharan Africa Total Diet Study in Benin, Cameroon, Mali and Nigeria Reveals the Presence of 164 Mycotoxins and Other Secondary Metabolites in Foods. *Toxins (Basel)* **2019**, *11*.
49. Salau, I.A.; Shehu, K.; Muhammad, S.; Umar, R.A.; Sciences, G.J.o.A. Aflatoxin Contamination of Stored Groundnut Kernel in Sokoto State, Nigeria. *Greener J Agr Sci* **2016**, *6*, 285–293.
50. Oloyede, M.; Williams, A.; Benson, N. Aflatoxin Contamination of Some Edible Grains from Lagos and Ota Markets, Nigeria. *Environ. Sci. Technol.* **2016**, *1*, 396–399.
51. Liverpool-Tasie, L.S.O.; Turna, N.S.; Ademola, O.; Obadina, A.; Wu, F. The occurrence and co-occurrence of aflatoxin and fumonisin along the maize value chain in southwest Nigeria. *Food Chem. Toxicol.* **2019**, *129*, 458–465.
52. Adebajo, L.O.; Idowu, A.A.; Adesanya, O.O. Mycoflora, and mycotoxins production in Nigerian corn and corn-based snacks. *Mycopathologia* **1994**, *126*, 183–92.
53. Hussaini, A.M.; Timothy, A.G.; Olufunmilayo, H.A.; Ezekiel, A.S.; Godwin, H.O. Fungi and some mycotoxins found in mouldy Sorghum in Niger State, Nigeria. *World Journal of Agricultural Sciences* **2009**, *5*, 5–17.
54. Njumbe Ediage, E.; Hell, K.; De Saeger, S. A comprehensive study to explore differences in mycotoxin patterns from agro-ecological regions through maize, peanut, and cassava products: A case study, Cameroon. *J. Agric. Food Chem.* **2014**, *62*, 4789–4797.
55. Agbetiameh, D.; Ortega-Beltran, A.; Awuah, R.T.; Atehnkeng, J.; Cotty, P.J.; Bandyopadhyay, R. Prevalence of Aflatoxin Contamination in Maize and Groundnut in Ghana: Population Structure, Distribution, and Toxigenicity of the Causal Agents. *Plant Dis.* **2017**, *102*, 764–772.
56. Hanvi, D.M.; Lawson-Evi, P.; De Boevre, M.; Goto, C.E.; De Saeger, S.; Eklu-Gadegbeku, K. Natural occurrence of mycotoxins in maize and sorghum in Togo. *Mycot. Res.* **2019**.

57. Dadzie, M.A.; Oppong, A.; Ofori, K.; Eleblu, J.S.; Ifie, E.B.; Blay, E.; Obeng –Bio, E.; Appiah-Kubi, Z.; Warburton, M.L. Distribution of Aspergillus flavus and aflatoxin accumulation in stored maize grains across three agro-ecologies in Ghana. *Food Control* **2019**, *104*, 91–98.
58. Wyk, P.S.v.; Merwe, P.J.A.V.d.; Subrahmanyam, P.; Boughton, D. Aflatoxin contamination of groundnuts in Mozambique. *International Arachis Newsletter* **1999**, *19*, 25–27.
59. Van Rensburg, S.J.; Cook-Mozaffari, P.; Van Schalkwyk, D.J.; Van der Watt, J.J.; Vincent, T.J.; Purchase, I.F. Hepatocellular carcinoma and dietary aflatoxin in Mozambique and Transkei. *Br. J. Cancer* **1985**, *51*, 713–726.
60. Rubel, F.; Kottek, M. World Maps of Köppen-Geiger Climate Classification. 2020. Accessed: 11 February 2020; Available from: http://koeppen-geiger.vu-wien.ac.at.
61. Reddy, K.R.; Reddy, C.S.; Muralidharan, K. Detection of *Aspergillus* spp. and aflatoxin B1 in rice in India. *Food Microbiol.* **2009**, *26*, 27–31.
62. Mohana, D.; Thippeswamy, S.; Abhishek, R.; Shobha, B.; Mamatha, M. Studies on seed-borne mycoflora and aflatoxin B1 contaminations in food based seed samples: Molecular detection of mycotoxigenic Aspergillus flavus and their management. *Int. Food Res. J.* **2017**, *24*, 422–427.
63. Bhat, R.V.; Vasanthi, S.; Rao, B.S.; Rao, R.N.; Rao, V.S.; Nagaraja, K.V.; Bai, R.G.; Prasad, C.A.K.; Vanchinathan, S.; Roy, R.; et al. Aflatoxin b1 contamination in maize samples collected from different geographical regions of India—a multicentre study. *Food Addit Contam: Part B* **1997**, *14*, 151–156.
64. Ratnavathi, C.V.; Komala, V.V.; Chavan, U.D. Chapter 3 - Mycotoxin Contamination in Sorghum. In *Sorghum Biochemistry*; Ratnavathi, C.V.; Patil, J.V.; Chavan, U.D., Eds.; Academic Press: San Diego, 2016, pp. 107–180.
65. Siruguri, V.; Kumar, P.U.; Raghu, P.; Rao, M.V.; Sesikeran, B.; Toteja, G.S.; Gupta, P.; Rao, S.; Satyanarayana, K.; Katoch, V.M.; et al. Aflatoxin contamination in stored rice variety PAU 201 collected from Punjab, India. *Indian J. Med. Res.* **2012**, *136*, 89–97.
66. Koirala, P.; Kumar, S.; Yadav, B.K.; Premarajan, K.C. Occurrence of aflatoxin in some of the food and feed in Nepal. *Indian J. Med. Sci.* **2005**, *59*, 331–6.
67. Quitco, R.T. Aflatoxin studies in the Philippines. in Fungi and mycotoxins in stored products: Proceedings of an international conference. Bangkok, Thailand: ACIAR Proceedings. 1991.
68. Arim, R.H. Mycotoxin contamination of food and feeds in the Philippines. *JSM Mycotoxins* **2004**, *2003*, 167–173.
69. Sales, A.C.; Yoshizawa, T. Updated profile of aflatoxin and Aspergillus section Flavi contamination in rice and its byproducts from the Philippines. *Food Addit Contam: Part B* **2005**, *22*, 429–36.
70. Panrapee, I.; Phakpoom, K.; Thanapoom, M.; Nampeung, A.; Warapa, M. Exposure to aflatoxin B1 in Thailand by consumption of brown and color rice. *Mycot. Res.* **2016**, *32*, 19–25.
71. Shank, R.C.; Gordon, J.E.; Wogan, G.N.; Nondasuta, A.; Subhamani, B. Dietary aflatoxins and human liver cancer. III. Field survey of rural Thai families for ingested aflatoxins. *Food Cosmet. Toxicol.* **1972**, *10*, 71–84.
72. Shank, R.C.; Wogan, G.N.; Gibson, J.B.; Nondasuta, A. Dietary aflatoxins and human liver cancer. II. Aflatoxins in market foods and foodstuffs of Thailand and Hong Kong. *Food Cosmet. Toxicol.* **1972**, *10*, 61–69.

73. Kooprasertying, P.; Maneeboon, T.; Hongprayoon, R.; Mahakarnchanakul, W. Exposure assessment of aflatoxins in Thai peanut consumption. *Cogent Food & Agriculture* **2016**, *2*.
74. Anukul, N.; Vangnai, K.; Mahakarnchanakul, W. Significance of regulation limits in mycotoxin contamination in Asia and risk management programs at the national level. *J. Food Drug Anal.* **2013**, *21*, 227–241.
75. Leong, Y.-H.; Ismail, N.; Latif, A.A.; Ahmad, R. Aflatoxin occurrence in nuts and commercial nutty products in Malaysia. *Food Control* **2010**, *21*, 334–338.
76. Khayoon, W.S.; Saad, B.; Lee, T.P.; Salleh, B. High performance liquid chromatographic determination of aflatoxins in chilli, peanut and rice using silica based monolithic column. *Food Chem.* **2012**, *133*, 489–496.
77. Semple, R.L.; Frio, A.S.; Hicks, P.A.; Lozare, J.V. Mycotoxin prevention and control in foodgrains 1991, Bankok, Thailand: FAO. Accessed: Available from: http://www.fao.org/3/X5036E/x5036E1b.htm#Mycotoxins%20in%20foodgrains%20in%20some%20Asian%20countries.
78. Arzandeh, S.; Selamat, J.; Lioe, H. Aflatoxin in raw peanut kernels Marketed in Malaysia. *J. Food Drug Anal.* **2010**, *18*, 44–50.
79. Soleimany, F.; Jinap, S.; Faridah, A.; Khatib, A. A UPLC–MS/MS for simultaneous determination of aflatoxins, ochratoxin A, zearalenone, DON, fumonisins, T-2 toxin and HT-2 toxin, in cereals. *Food Control* **2012**, *25*, 647–653.
80. Reddy, K.R.; Farhana, N.I.; Salleh, B. Occurrence of *Aspergillus* spp. and aflatoxin B1 in Malaysian foods used for human consumption. *J. Food Sci.* **2011**, *76*, T99–104.
81. Do, T.H.; Tran, S.C.; Le, C.D.; Nguyen, H.-B.T.; Le, P.-T.T.; Le, H.-H.T.; Le, T.D.; Thai-Nguyen, H.-T. Dietary exposure and health risk characterization of aflatoxin B1, ochratoxin A, fumonisin B1, and zearalenone in food from different provinces in Northern Vietnam. *Food Control* **2020**, *112*, 107108.

© 2020 by the authors. Licensee MDPI, Basel, Switzerland. This article is an open access article distributed under the terms and conditions of the Creative Commons Attribution (CC BY) license (http://creativecommons.org/licenses/by/4.0/).
